# Platelet membrane decorated exosomes enhance targeting efficacy and therapeutic index to alleviate arterial restenosis

**DOI:** 10.7150/thno.103747

**Published:** 2025-01-01

**Authors:** Shan Lu, Ruihan Wang, Minghao Cai, Chen Yuan, Bin Gao, Daqiao Guo, Yisheng Xu, Weiguo Fu, Xiaohua Yu, Yi Si

**Affiliations:** 1 Department of Vascular Surgery, Zhongshan Hospital Fudan University, Shanghai, 200032, PR China.; 2 Institute of Vascular Surgery, Fudan University, Shanghai, 200032, PR China.; 3 National Clinical Research Center for Interventional Medicine, Shanghai, 200032, PR China.; 4 State Key Laboratory of Chemical Engineering, East China University of Science and Technology, Shanghai, 200237, PR China.; 5 Department of Orthopedics, The second Affliated Hospital of Zhejiang University School of Medicine, Hangzhou, 310009, Zhejiang, PR China.; 6 Key Laboratory of Motor System Disease Research and Precision Therapy of Zhejiang Province, Hangzhou, 310009, Zhejiang, PR China.

**Keywords:** arterial restenosis, MSC exosomes, platelet-mimetic, targeted delivery, endothelial repair

## Abstract

**Rationale:** Postinterventional restenosis is a major challenge in the treatment of peripheral vascular disease. Current anti-restenosis drugs inhibit neointima hyperplasia but simultaneously impair endothelial repair due to indiscrminative cytotoxity. Stem cell-derived exosomes provide multifaceted therapeutic effects by delivering functional miRNAs to endothelial cells, macrophages, and vascular smooth muscle cells (VSMCs). However, their clinical application is severly limited by poor targeting and low tissue uptake in injured vessel.

**Methods:** To address this challenge, we constructed platelet-mimetic exosomes (PM-EXOs) by fusing mesenchymal stem cell (MSC)-derived exosomes with platelet membrane in order to harness the natural ability of platelets to target vascular injury, evade clearance by the mononuclear phagocyte system, and penetrate into the intima by hitchhiking on inflammatory monocytes.

**Results:** PM-EXOs demonstrated enhanced cellular uptake by endothelial cells and macrophages, exerting proangiogenic and immunomodulatory effects via the delivery of functional miRNAs* in vitro*. The intravenously administrated PM-EXOs exhibited extended circulation time and a 4-fold enhancement in targeting injured arteries compared to unmodified exosomes. In mouse and rat carotid artery injury models, PM-EXOs were shown to promote endothelial repair on the denuded arterial wall, lower the M1/M2 ratio of infiltrated macrophages, and eventually inhibit phenotypic switch of vascular smooth muscle cells and reduce the formation of neointima without causing systemic toxicity.

**Conclusions:** This biomimetic strategy may be leveraged to boost the therapeutic index of exosomes and realize the multifaceted treatment of arterial restenosis.

## Introduction

Restenosis is a major obstacle in the management of peripheral arterial diseases, often leading to treatment failure and the need for reintervention following endovascular surgery 1, 2. This pathological process is initiated by endothelial disruption and exaberated by neointima hyperplasia, which involves inflammatory cell infiltration, vascular smooth muscle cell (VSMC) migration, and collagen deposition 3. In clinic setting, antirestenotic drugs such as paclitaxel or limus-based therapeutics, are deployed on stents and balloons to target proliferative VSMCs and prevent neointima formation. However, the indiscriminate cytotoxicity of antirestenotic drugs also impairs endothelial cells and retards the crucial process of re-endothelialization 4, 5. This delayed endothelial recovery exposes the intima to persistant external stimuli and inflammatory envinroments, triggering unregulated VSMC proliferation and migration. In turn, the thickened intima further hinders endothelial recovery, creating a vicious cycle that increases the risk of late-stage restenosis and thrombosis 6, 7. Therefore, there is an urgent need for innovative, multitargeted strategies that concurrently promote endothelial repair and inhibit neointima formation.

In recent years, various therapies, including drug delivery, gene vectors, and peptide vaccines, have been proposed for treating postinterventional arterial restenosis 8-10. Among these, stem cell-derived exosome therapy is a particularly promising strategy due to its angiogenic potential, immunomodulatory capacity, and regenerative action 11, 12. It has been demonstrated that exosomes secreted by mesenchymal stem cells (MSCs) are able to promote endothelial cell proliferation, rejuvenate senescent endothelial progenitor cells, and modulate the immune microenvironment by delivering functional payloads such as proteins and microRNAs (miRNAs) to neighboring cells 13, 14. For instance, Cheng et al. decreased in-stent restenosis in rats by implanting MSC exosome-eluting stents in their abdominal aorta, as MSC-derived exosomes inhibited VSMC migration and regulated local inflammation 15. However, several challenges currently limit the clinical application of exosome-based therapies: i) absence of targeting ability leads to a constrained accumulation of exosomes at the lesion site 16, ii) poor tissue permeability and high washout rates results in limited exosome penetration into the intima to affect VSMCs 17, and iii) rapid clearance by the mononuclear phagocyte system signicantly diminishes exosome bioavailability after systemic administration 18. According to Wiklander et al., functionally effective exosomes account for less than 3% of the injected volume 19, severely constraining their restorative potential and rendering therapeutic benefits irreproducible. Hence, for treating restenosis, it is imperative to develop exosome engineering strategies that can achieve selective targeting and efficient cargo delivery to maximize the comprehensive therapeutic effects of exosome therapy.

After arterial injury, platelets are rapidly activated and efficiently accumulate at the lesion site in a self-amplified manner. The innate anchoring of platelets to injured arteries is primarily mediated by the interaction between platelet membrane protein GPIbα and von Willebrand Factor (vWF) secreted by the injured endothelium 20, 21. Mimicking platelet functionality could thus enhance the concentration of exosomes at target arteries and improve delivery efficiency for restenosis treatment. Moreover, platelets provide a “don't-eat-me” signal by expressing immunomodulatory protein CD47, allowing them to evade clearance by the mononuclear phagocyte system and prolong circulation 22. As endothelial repair progresses and cell junctions stabilize, the tissue permeability of the injured arterial wall decreases, significantly narrowing the therapeutic window for restenosis 23. Studies have shown that platelets can aggregate with immune cells such as monocytes, neutrophils, and eosinophils after arterial injury and migrate to the lesion site together 24, 25. Specifically, platelets have been reported to cross the endothelium by hitching onto peripheral monocytes, achieving enhanced permeation and retention effects 26. Leveraging this platelet deliveiry mechanism could facilitate the efficient delivery of nanomedicines deep into tissues, enabling therapeutic effects across different severities and stages of restenotic lesions. Recent studies have introduced platelet membrane and its targeting proteins onto different nanoparticles in several diseases, including myocardial infarction, venous thrombosis, and cancer 27-30. Given the inherent targeting, immune evasion, and tissue permeability properties of platelets, engineering exosomes with platelet membrane could be expected to efficiently direct exosomes to the injured arterial wall and optimize therapeutic effects.

**Scheme 1:**
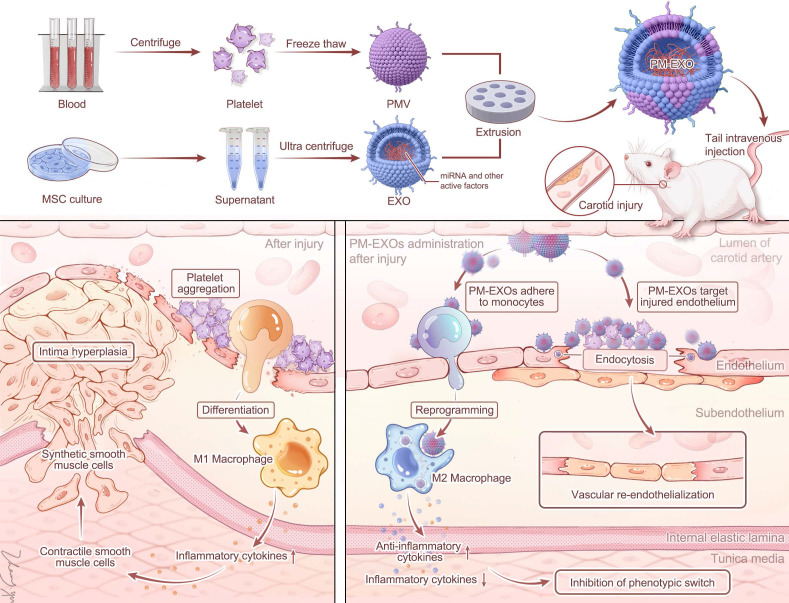
Schematic diagram of PM-EXO fabrication and the therapeutic effects in restenosis treatment. PM-EXOs selectively target arterial injury and exert multifaceted synergistic effects on endothelial repair, inflammatory regulation, and inhibition of neointima formation.

Based on the considerations outlined above, we developed platelet-mimetic exosomes (PM-EXOs) to harness the natural targeting properties of platelet while preserving the therapeutic effects of exosomes. The construction of PM-EXOs was achieved through the hybridization of MSC-EXOs and platelet membrane vesicles (PMVs) using a mechanical fusion-extrusion method (**Scheme 1**). The physiochemical properties, targeting ability, cargo-delivery efficiency, and proangiogenic and immunomodulatory potential of PM-EXOs were thoroughly evaluated *in vitro* and in a mouse carotid artery wire injury model and a rat carotid artery balloon injury model. Importantly, PM-EXOs demonstrated outstanding therapeutic efficacy for arterial restenosis through the following synergistic effects: i) accelerated endothelial recovery, ii) alleviation of arterial wall inflammation by macrophage repolarization, and iii) reduction of neointima hyperplasia by inhibiting VSMC proliferation and phenotypic switch. Collectively, our study introduced multifunctional platelet-mimetic EXOs as a promising strategy for the targeted delivery and comprehensive treatment of arterial restenosis.

## Methods

### Materials

Mini extruder and polycarbonate porous membranes were obtained from Avanti Polar Lipids (Alabaster, AL, USA). FRET dyes rhodamine DHPE and NBD-PE were obtained from Invitrogen (Thermo Fisher Scientific, Waltham, MA, USA), as were fluorescent dyes DiO (3,3-dioctadecyloxacarbocyanine perchlorate), DiI (1,1^'^-dioctadcyl-3,3,3^,^,3^,^-tetramethylindocarbocyanine perchlorate), and DiD (1,1-dioctadecyl-3,3,3^,^,3^,^-tetramethylindodicarbocyanine, 4-chlorobenzenesulfonate salt). Lipopolysaccharides (LPS) and Evans blue dye were purchased from Sigma-Aldrich (St. Louis, MO, USA). Antibodies for flow cytometry analysis were purchased from BioLegend (San Diego, CA, USA), and antibodies for western blotting analysis were purchased from Abcam (Cambridge, MA, USA). The suppliers of other reagents and equipment are specified below.

### Cell lines

HUVEC, RAW 264.7, A7r5 and THP-1 cell lines were purchased from the American Type Culture Collection (ATCC, Manassas, VA, USA). HUVEC, Raw 264.7 cells or A7r5 cells were cultured in Dulbecco's Minimal Essential Medium (DMEM) containing 10% fetal bovine serum (FBS), 1% GlutaMax-1, and 1% penicillin and streptomycin at 37 ℃ in a 5% CO_2_ environment. THP-1 cells were cultured in RPMI 1640 complete medium. Rat bone marrow MSCs were obtained from the Cell Bank of Chinese Academy of Sciences and cultured in MSC medium.

### Preparation of PM-EXOs

Platelets were isolated from human O^-^ type blood plasma. After repeated freeze-thaw cycles and an 8000 × g centrifugation for 15 minutes, platelet pellets were resuspended to obtain PMVs. Bone marrow-derived mesenchymal stem cells (BMSCs) were cultured in MSC medium, and the supernatants were ultracentrifugated at 100,000 × g to isolate MSC-derived EXOs. Following this, EXOs were passed through a 220-μm filter to remove impurities. A bicinchoninic acid (BCA) protein assay kit (Sigma-Aldrich) was used to quantify the protein weight of PMVs and EXOs, after which PMVs and EXOs were mixed at varying concentration ratios and subjected to sonication at a frequency of 42 kHz and 100 W for 3 minutes. To prepare the PM-EXO biomimetic system, the sonicated mixture was sequentially extruded 10 times through 1-μm, 400-nm, and 200-nm polycarbonate porous membranes using an Avanti Mini Extruder.

### Membrane fusion verification

Förster resonance energy transfer (FRET) was employed to confirm membrane fusion by measuring fluorescence intensity and to evaluate the fusion efficiency of PMVs and EXOs at different mixing ratios. PMVs were first labeled with rhodamine DHPE (FRET acceptor; excitation/emission maxima = 560/580 nm) and NBD-PE (FRET donor; excitation/emission maxima = 463/536 nm) and then mixed with EXOs at different ratios (PMV:EXO) of 2:0, 2:1, 2:2, 2:4, and 2:6. PM-EXO samples of different mixing ratios were examined with an Infinite 200 PRO microplate reader (Tecan, Männedorf, Switzerland), and the fluorescence spectrum spanning from 500 to 700 nm was collected with an excitation wavelength of 470 nm. The morphological characteristics and physical properties of PM-EXOs, including particle size and membrane zeta potential, were observed and assessed with an H-600 transmission electron microscope (TEM; Hitachi, Tokyo, Japan) and a Zetasizer Pro (Malvern Panalytical, Malvern, UK). EXOs and PMVs were also prepared as the controls. For serum stability examination, PM-EXOs and EXOs were separately incubated in 10% human serum for 4 h, and the absorbance of the suspension was measured at 30-min intervals by Multiskan Skyhigh microplate spectrophotometer (Thermo Fisher Scientific).

To confirm the colocalization of membranes, PMVs were labeled with DiO (excitation/emission maxima = 484/501 nm) while EXOs were labeled with DiI (excitation/emission maxima = 549/565 nm) at 37 ℃ for 30 min. After centrifugation and resuspension to remove excess dye, PMVs labeled with green dye and EXOs labeled with red dye underwent membrane fusion, and the overlap of fluorescence was examined under a fluorescence microscope.

In order to assess the integration of cell membrane proteins, western blotting was employed to detect the expression of platelet-specific (TSG101, CD81, CD63, CD9) and exosome-specific (GPIba, GPVI, CD47, CD42a) membrane proteins in the PMV, EXO, and PM-EXO groups. In addition to western blotting, the Coomassie brilliant blue method was also used to measure the total protein content of PMVs, EXOs, and PM-EXOs.

### MiRNA sequencing of PM-EXOs

Total RNA was respectively extracted from EXOs and PM-EXOs, purified using a miRNeasy Serum/Plasma Kit (Qiagen, Hilden, Germany), and subjected to quality inspection in a Bioanalyzer 2100 (Agilent, Santa Clara, USA). RNA molecules ranging from 18 to 30 nt were selectively concentrated through polyacrylamide gel electrophoresis. The concentrated miRNA was reverse transcribed into complementary DNA (cDNA) using the QIAseq miRNA Library Kit (Qiagen), and the products were then enriched with PCR to create the final cDNA library. Clusters were generated using cBot with the library diluted to 10 pM and were then sequenced on a HiSeq X Ten system (Illumina, San Diego, CA, USA). The library construction and sequencing were performed by Sinotech Genomics Co., Ltd (Shanghai, China). Differential expression analysis to filter miRNAs with a *P* value < 0.05 was performed using DESeq software (Bioconductor), and miRNA target genes searching was performed using the “multimiR” package. Following this, GO analysis and Kyoto Encyclopedia of Genes and Genomes (KEGG) pathways analysis were respectively performed via the “enrich” R package (The R Foundation for Statistical Computing). MiRNAs with abundant expression related to angiogenesis or immunomodulatory functions were screened.

### *In vitro* targeting and miRNA delivery of PM-EXOs

HUVECs, RAW 264.7 cells, and A7r5 cells were seeded onto 12-well plates (10^5^ per well) and placed in respective cell culture incubators overnight. PBS or 25 ug of DiI-labeled EXOs or PM-EXOs were added to each well and cocultured at different times (1 h, 2 h, 4 h, and 6 h). After incubation, cells were rinsed to remove excess dye and collected for flow cytometry analysis to evaluate cellular uptake. FlowJo 10.8.1 software (BD, Franklin Lakes, NJ, USA) was used to calculate the uptake rate of EXOs and PM-EXOs and determine the coculture timepoint at which the EXO group and PM-EXO group showed the most pronounced difference in cellular uptake. Confocal laser scanning microscopy (CLSM) was then employed to visualize the uptake of EXOs and PM-EXOs in different cells. HUVECs were immunofluorescence-stained with anti-vWF antibody at 4 ℃ overnight and then incubated with secondary antibody at 37 ℃ for 1 h. Nuclei were counterstained with DAPI for 10 min, and then cells were subjected to CLSM. To investigate the ability of PM-EXOs to deliver miRNAs to target cells, HUVECs and RAW 264.7 cells were cocultured with PBS, EXO, and PM-EXOs for 24 h. Subsequently, quantitative real-time polymerase chain reaction (qRT-PCR) was used to assess the intracellular expression of the top three miRNAs associated with angiogenesis and immunomodulatory function acquired from miRNA sequencing.

### Functional analysis of PM-EXOs *in vitro*

To investigate the ability of PM-EXOs to promote angiogenesis, HUVECs were seeded onto 12-well plates in endothelial cell medium (ECM) and treated with PBS, EXOs, and PM-EXOs (25 ug/per well) for 6 h. After additional 48-h incubation, the cell proliferation levels were measured using an EdU assay kit (C10339; Invitrogen). Western blotting assay was used to evaluate the expression levels of vWF and VE-cadherin, which can reflect the migration and proliferation capacity of HUVECs. The cellular expression of these two indicators was also measured with immunofluorescence staining. Scratch assays were conducted to evaluate cell migration capacity. HUVECs were coincubated with PBS, EXOs, or PM-EXOs for 24 h, and the cell migration rate was analyzed with ImageJ 22.3.0 software (National Institutes of Health, Bethesda, MD, USA). Additionally, tube formation assays were performed, in which HUVECs were treated with PBS, EXOs, or PM-EXOs for 6 h and observed using an inverted microscope. The tube length and number of nodes were measured with ImageJ 22.3.0 software. To investigate the mechanism by which PM-EXOs promote angiogenesis, western blotting was used to assess the expression level of VEGF-VEGFR2 signaling pathway with anti-VEGF antibody and anti-VEGFR2 antibody.

With respect to the inflammatory regulation ability of PM-EXOs, RAW 264.7 cells were seeded in 12-well plates in DMEM and treated with 50 ng/ml of LPS for 24 h to simulate an inflammatory environment. The culture medium was then changed, and the cells were respectively treated with PBS, EXOs, PM-EXOs, or PMVs (25 ug/per well) for 24 h. The control group received treatment with culture medium but not with LPS or other treatment. The cells were collected and stained with phycoerythrin (PE)-conjugated CD86 and fluorescein isothiocyanate (FITC)-conjugated CD206, which are respectively specific indicators for proinflammatory M1- and anti-inflammatory M2-type macrophages. Flow cytometry was used to investigate the phenotypic switch of macrophages. Moreover, the expression of iNOS is another indicator for M1 macrophages, and western blotting was performed to measure the expression level of iNOS and CD206 in the different groups. RAW 264.7 cells were also immunofluorescence-stained with anti-iNOS antibody and anti-CD206 antibody and observed using CLSM. The concentration of proinflammatory cytokines (TNF-α) and anti-inflammatory cytokines (IL-10) in supernatants was detected using enzyme-linked immunosorbent assay (ELISA) kits (BioLegend) following the manufacturer's protocol. At the gene level, the expression of proinflammatory cytokines (*TNF-α*, *IL-1β*) and anti-inflammatory cytokines (*TGF-β*, *Arg-1*) was also measured using qRT-PCR analysis. To investigate the mechanism of the PM-EXO immunoregulation effect, proteins were extracted from all samples and incubated with anti-PTEN antibody, anti-AKT antibody and anti-p-AKT antibody. The amount of target proteins was quantified using ImageJ gray scanning and normalized against the expression of β-actin.

Regarding the effect of inhibiting neointima hyperplasia, an indirect coculturing method was used, in which A7r5 cells were incubated with cell culture supernatant obtained from RAW 264.7 cells treated under different conditions for 24 h. Western blotting was performed to assess the phenotypic switch between contractile and synthetic VSMCs with anti-OPN antibody, anti-α-SMA antibody, anti-CNN-1 antibody, and anti-SM22-α antibody. The expression of these indicators was also evaluated at the gene level using qPCR assay. Additionally, A7r5 cells were immunofluorescence-stained with anti-α-SMA antibody and observed using CLSM. Cell functional assays, including scratch assay and transwell assay, were conducted to assess the migration ability of A7r5 cells. Briefly, 10^5^ A7r5 cells were seeded in the upper chamber of an 8-μm transwell plate. PBS or 25 ug of EXOs or PM-EXOs were then added to the medium for 24 h. Subsequently, A7r5 cells in the lower chamber were collected, and the cell intensity was measured.

### Mouse carotid artery wire injury

A mouse carotid artery wire injury model was created using 6 to 8-week-old male mice. All *in vivo* experiments in this study were performed according to the guidelines of the Animal Ethics Committee of Zhongshan Hospital Fudan University (Shanghai, China). Briefly, the mice were anesthetized via an intraperitoneal injection of 0.15 ml of 0.8% pentobarbital sodium. A 1-cm skin incision was made in the median region of the neck area, and the left common carotid artery (LCCA), left external carotid artery (ECA), and left internal carotid artery (ICA) were isolated. The proximal end of the ECA was ligated, after which the blood flow of the CCA and ICA was temporarily blocked with silk slipknots composed of 0/6 silk sutures. Next, a small incision was made distal to the ECA, and a curved flexible wire (0.38 mm) was inserted into the CCA via the transverse arteriotomy. The wire was passed along the vessel five times to achieve endothelial denudation, with the force of the passage movement being the same for each mouse to enhance experimental reproducibility. The skin incision was then closed. Subsequently, 3, 7, or 28 days after injury, the mice were killed, and the LCCAs were harvested for further experiments.

### Rat carotid artery balloon injury

Male Sprague-Dawley rats weighing 200-250 g were used for the carotid artery balloon model. Initially, a small incision was made on top of the trachea, and the perivascular adipose tissue was excised. Subsequently, a balloon catheter 1.5 mm in diameter was inserted into the LCCA via the arteriotomy on the CEA. The balloon catheter was then inflated and pulled back and forth through the LCCA five times with constant rotation. After the recovery of blood flow, the skin incision was closed. Fourteen days after injury, all rats were killed, and their carotid arteries were harvested for experiments.

### Biodistribution and targeting profile of PM-EXOs

To evaluate the pharmacodynamics of EXOs and PM-EXOs *in vivo*, mice were intravenously injected with EXOs or PM-EXOs (100 ug protein/per mouse) which had been labeled with DiD far-red plasma membrane fluorescent probe (excitation/emission maxima = 644/665 nm). The blood was then collected from the retro-orbital sinus of mice at predetermined timepoints (30 min, 1 h, 2 h, 4 h, 6 h, 24 h, and 48 h) and measured with an *In Vivo* Imaging System (IVIS) Lumina III system (PerkinElmer, Waltham MA, USA). For assessing the targeting ability of PM-EXOs *in vivo*, DiD-labeled EXOs and PM-EXOs (100 ug protein/per mouse; 300 ug protein/per rat) were injected via the caudal vein 1 day after the establishment of the carotid injury model. At 24 h postinjection, the mice were killed, and the injured carotid artery and other important organs (heart, liver, spleen, lung, kidney, brain) were harvested and placed under the IVIS system for *ex vivo* imaging and fluorescence intensity measurement.

### Treatment protocol and the *in vivo* therapeutic effects of PM-EXOs

The mice were randomly assigned to three groups 24 h after carotid injury and injected with PBS, EXOs, or PM-EXOs (200 ug protein/per mouse). Mice in the sham group served as the negative control and did not receive carotid surgery or any injection.

To assess the re-endothelialization effect of PM-EXOs, the mice were killed on day 3 and day 7, and the carotid arteries were harvested for Evans blue staining. Briefly, a 200-ul solution of 0.5% Evans blue dye was intravenously administered to the mice and allowed to circulate in the bloodstream for 10 min. Following this, 2 ml of 0.9% saline was perfused to remove unbounded dye and the carotid arteries were collected. Images were obtained, and the stained endothelial-denuded area was measured using ImageJ software. Additionally, the carotid arteries harvested on day 7 were sliced and immunofluorescence-stained with anti-CD31 antibody.

The mice harvested on day 3 and day 7 were also used to evaluate the infiltration and phenotypic switch of inflammatory cells at the site of injury. The carotid arteries were immunofluorescence stained with anti-iNOS antibody and anti-CD206 antibody. Additionally, ELISA was performed to measure the levels of TNF-α, IL-1β, TGF-β, and IL-10 in homogenized carotid artery tissues and that of TNF-α of the whole blood samples.

For the assessment of neointima hyperplasia, the mice were killed on day 28, and their carotid arteries were fixed in 4% paraformaldehyde, embedded, and sectioned for subsequent staining procedures. Hematoxylin and eosin (H&E) immunostaining was performed, and the areas of the intima and media were measured with ImageJ software. To investigate the phenotypic switch of VSMCs, the sections of injured carotid arteries were also immunofluorescence-stained with anti-α-SMA antibody and anti-OPN antibody and observed using CLSM. Additionally, the expressions of OPN, α-SMA, CNN-1, SM22-α in mouse carotid artery tissues assessed via western blotting.

### Statistical analysis

All data are presented as the mean ± SD and were visualized using GraphPad Prism 10.0.3 software (GraphPad Software, Inc., La Jolla, CA, USA). Comparisons between groups were performed using the Student *t* test or one-way analysis of variance in SPSS 29.0.1 software (IBM Corp., Armonk, NY, USA). A P value < 0.05 was considered to indicate a significant difference.

## Results and Discussion

### Fabrication and characterization of PM-EXOs

**Figure 1:**
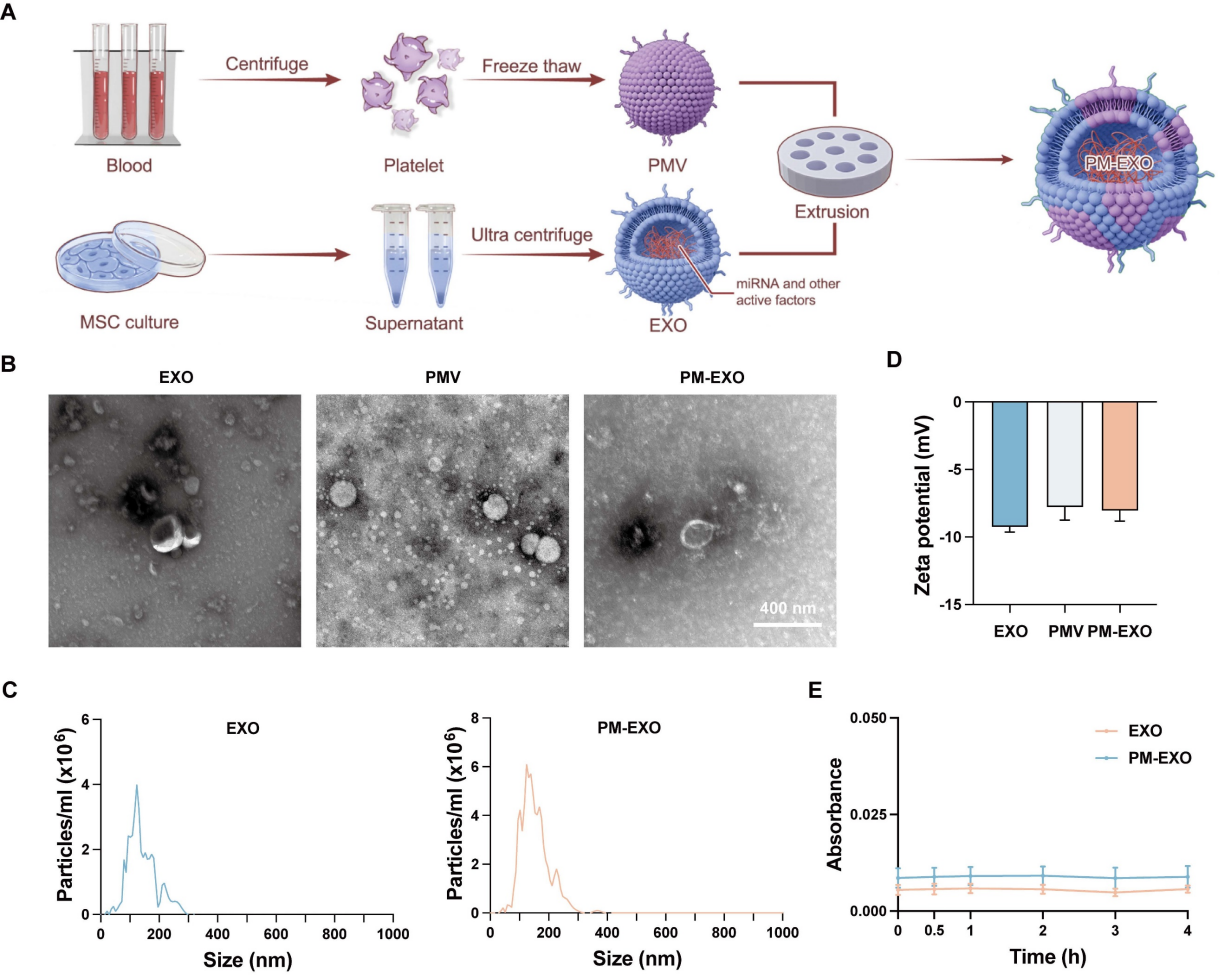
Fabrication and characterization of PM-EXOs. (A) Schematic illustration of PM-EXO fabrication. (B) Representative transmission electron micrographs of EXOs, PMVs, and PM-EXOs. Scale bar = 400 nm. (C) Particle size distribution of EXOs and PM-EXOs according to nanoparticle tracking analysis. (D) Surface zeta potential of EXOs, PMVs, and PM-EXOs. (E) Serum stability of EXOs and PM-EXOs monitored for 4 h. Data are expressed as the mean ± SD.

Bone marrow-derived MSCs were cultured, and EXOs were isolated using the ultrafiltration method as previously described 31. PMVs were obtained from human platelets through a repeated freeze-thaw process. Subsequently, PM-EXOs were fabricated by fusing EXOs and PMVs through serial mechanical extrusion (**Figure 1**A). Transmission electron microscope (TEM) images showed both EXOs and PM-EXOs had a round or elliptical shape with a smooth, uniform surface (Figure 1B), indicating that the vesicle structure was preserved during extrusion. Nanoparticle tracking analysis (NTA) revealed that PM-EXOs had a peak particle size of 119.4 ± 15.8 nm, a slight increase of 4.6 nm compared to EXOs (Figure 1C). The zeta potential of PM-EXOs was -8.1 ± 0.8 mV, intermediate between EXOs and PMVs, suggesting successful alteration in the membrane contents of the vesicles (Figure 1D). In addition, serum stability tests showed that the absorption of both EXOs and PM-EXOs remained stable in 10% human plasma over 4 h, indicating that PM-EXOs did not aggregate* in vivo* as EXOs (Figure 1E). Collectively, the assessments of physical properties demonstrated that PM-EXOs maintained intact vesicle structure and uniform nanoscale particle size with good serum stability, making them suitable for intravenous administration.

### Membrane fusion verification and cargo integrity assessment

**Figure 2:**
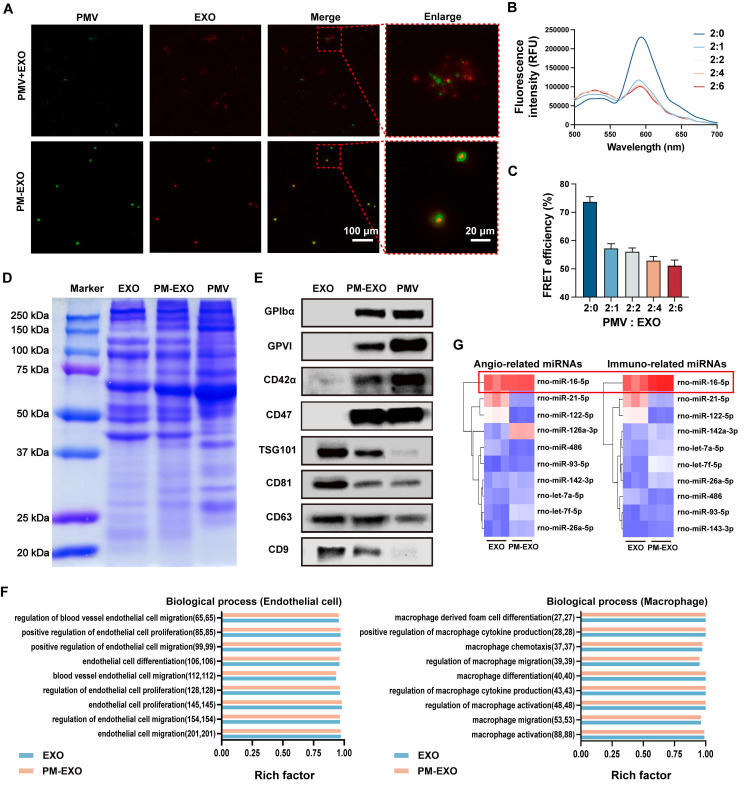
Verification of membrane fusion and assessment of the protein and miRNA profiles in PM-EXOs. (A) Representative immunofluorescence images of the PM-EXO and PMV + EXO mixture (green: PMV; red: EXO). Scale bar = 100 μm and scale bar = 20 μm (enlarged). (B) Fluorescence intensity after membrane fusion at different PMV:EXO ratios. PMVs were labeled with a pair of FRET dyes and extruded with increasing amounts of EXOs. (C) Quantification of FRET efficiency. (D) Protein profile of PM-EXOs assessed by Coomassie Blue staining. (E) Marker proteins in EXOs, PM-EXOs and PMVs detected by western blotting. (F) Gene enrichment analysis of biological process involved in endothelial cells and macrophages. (G) Heat map of angiogenesis-related miRNAs and immunomodulation-related miRNAs in EXOs and PM-EXOs (blue represents low expression and red represents high expression). Data are expressed as the mean ± SD (n = 3).

The membrane fusion of EXOs and PMVs was confirmed through immunofluorescence staining assay. PMVs were labeled with DiO (green), EXOs were labeled with DiI (red), and then the two stained samples underwent simple mixing or extrusion. Distinct green and red signals were observed in the mixed sample, while fused PM-EXOs displayed overlapping yellow fluorescence, confirming successful fusion of PMVs and EXOs (**Figure 2**A). To determine the optimal PMV:EXO ratio for membrane fusion, PMVs were labeled with a pair of FRET dyes and then extruded with EXOs at protein weight ratios of 2:0, 2:1, 2:2, 2:4, and 2:6. Increasing the EXO proportion led to a recovery of fluorescence and a decrease at 580 nm, indicating reduced FRET interaction due to membrane interspersion (Figure 2B). A 22.3% decrease in FRET deficiency at a 2:1 ratio confirmed adequate fusion, though higher EXO proportions caused only minor additional declines (Figure 2C). Therefore, a protein weight ratio of 2:1 was adopted for the fusion of PMVs and EXOs in subsequent experiments.

Cargo integrity of PM-EXOs was assessed by protein and miRNA profiling. Coomassie brilliant blue staining revealed a protein profile of PM-EXOs that encompassed both EXOs and PMVs (Figure 2D). Western blotting analysis further revealed the positive expression of EXO-associated proteins TSG101, CD81, CD63, and CD9, along with platelet-associated adhesion proteins GPIbα, GPVI, CD47, and CD42α in PM-EXOs (Figure 2E). These adhesion molecules are known to facilitate platelet aggregation at sites of endothelial injury. Therefore, the membrane fusion and transfer of membrane proteins potentially endowed PM-EXOs with the ability to selectively target injured arteries. Small RNA sequencing of EXOs and PM-EXOs revealed similar miRNA profiles (Figure S1) and gene enrichments in angiogenesis and immunomodulation-related processes (Figure S2 and Figure 2F). These results suggested that the membrane fusion process did not significantly alter the miRNA profile, implying that PM-EXOs maintain functional similarities to EXOs in modulating endothelial cells and macrophages. Notably, key miRNAs involved in angiogenesis and immunomodulation, such as miR-16-5p, were identified and found to be comparably expressed in both EXOs and PM-EXOs (Figure 2G).

Taken together, these findings confirmed that the membrane fusion process successfully introduced platelet membrane and its targeting proteins onto the surface of EXOs while retaining the cargo integrity of EXOs, with only slight changes in the miRNA profile.

### Promoted cellular uptake and miRNA delivery efficiency of PM-EXOs *in vitro*

**Figure 3:**
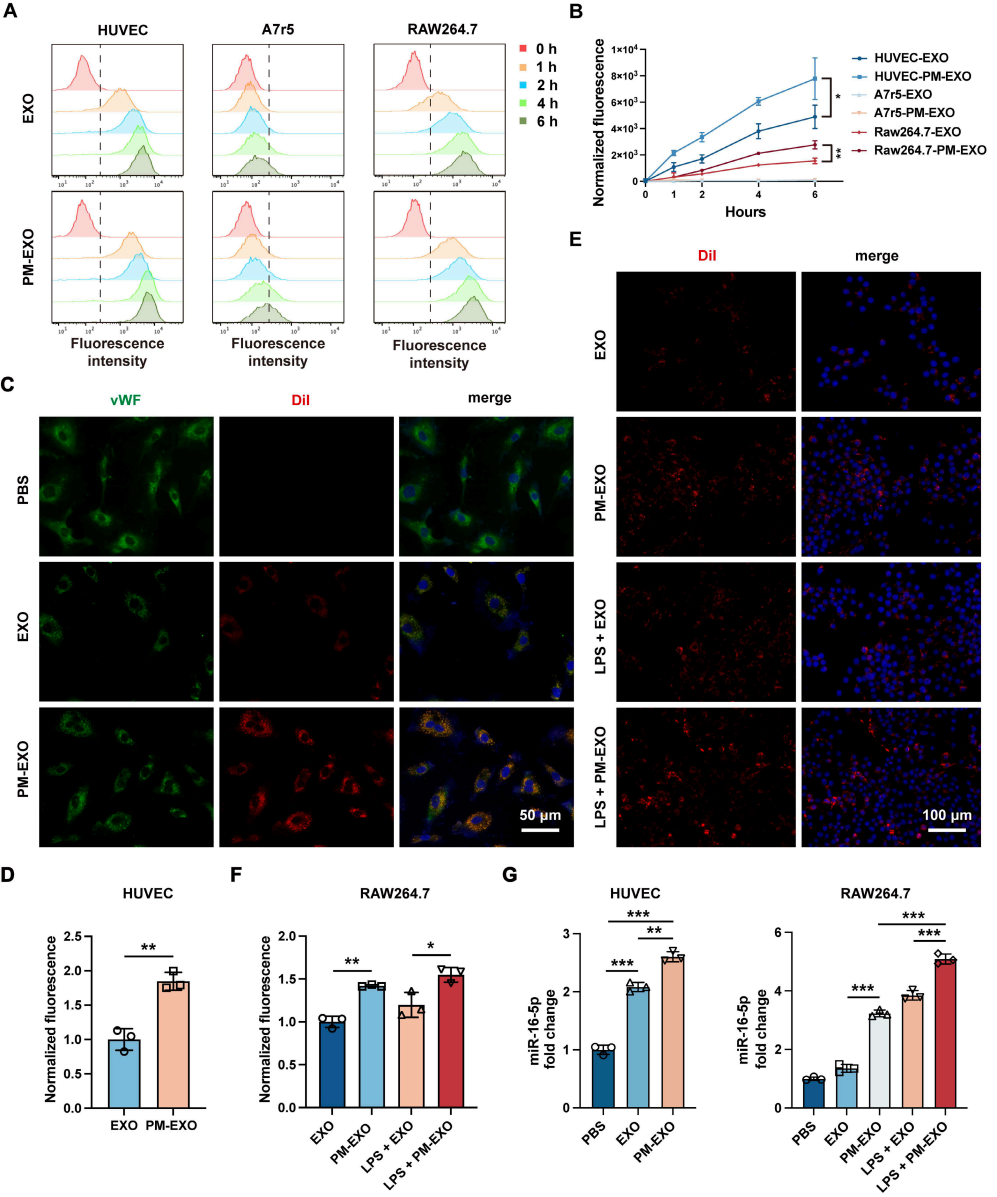
Cellular uptake and miRNA delivery ability of PM-EXOs. (A) Flow cytometry and (B) quantification of the normalized fluorescence intensity of DiI-labeled EXOs or PM-EXOs internalized by HUVECs, A7r5 cells, and RAW 264.7 cells at different timepoints (0, 1, 2, 4, and 6 h). (C) Representative fluorescence images and (D) semi-quantification of DiI-labeled EXOs or PM-EXOs (red) coincubated with HUVECs (green) for 6 h. Scale bar = 50 μm. (E) Representative fluorescence images and (F) semi-quantification of the fluorescence intensity of cellular uptake of DiI-labeled EXOs or PM-EXOs (red) by RAW 264.7 cells (blue) with or without LPS pretreatment. Scale bar = 100 μm. (G) Quantification of miR-16-5p in HUVECs and RAW264.7 cells after coincubation with EXOs or PM-EXOs for 24 h with or without LPS pretreatment. Data are expressed as the mean ± SD (n = 3, *P < 0.05, **P < 0.01, ***P < 0.001 by unpaired two-tailed Student's *t*-test (B, D) or by one-way ANOVA with a Tukey post hoc test (F, G)).

Injured arterial tissue primarily consists of endothelial cells, VSMCs, and infiltrating macrophages, which represent potential target cells for PM-EXOs 32. To demonstrate the targeting ability of PM-EXOs *in vitro*, the uptake of DiI-labeled EXOs and PM-EXOs by Human umbilical vein endothelial cells (HUVECs), A7r5 VSMCs and RAW 264.7 macrophages were examined at 1 h, 2 h, 4 h, and 6 h postincubation. As shown in **Figure 3**A, flow cytometry revealed a time-dependent increase in cellular uptake. A7r5 cells exhibited negligible uptake of both MSC-derived EXOs and PM-EXOs, while HUVECs and RAW 264.7 cells had uptake rates of PM-EXOs approximately 1.6-fold and 1.8-fold higher than those of EXOs, respectively (Figure 3B). Immunofluorescence staining corroborated these findings, showing more efficient internalization of DiI-labeled PM-EXOs by HUVECs compared to EXOs (Figure 3C-D). In order to better evaluate the targeting ability of PM-EXOs for activated macrophages at the site of injured artery, RAW 264.7 cells were pretreated with LPS for activation and then coincubated with EXOs or PM-EXOs. Uptake by activated RAW 264.7 cells increased for both EXOs and PM-EXOs, with PM-EXOs showing significantly higher uptake efficiency than EXOs regardless of LPS stimulation (Figure 3E-F).

To examine whether PM-EXOs could successfully deliver miRNAs to endothelial cells and macrophages, the expression of major angiogenesis-related miRNAs in HUVECs and immunomodulation-related miRNAs in RAW 264.7 cells was assessed via qRT-PCR according to the results of small RNA sequencing. Coincubation with EXOs or PM-EXOs significantly increased miR-16-5p expression in both cell types compared to PBS treatment, with PM-EXOs inducing a greater increase (Figure 3G). PM-EXOs also elevated the expression levels of other critical angio-miRNAs and immune-miRNAs (Figure S3). Notably, RAW 264.7 cells pretreated with LPS exhibited a more significant alteration in cellular miRNA levels after coincubation. This could be attributed to the LPS-induced upregulation of adhesive molecules on the macrophage surface that thereby enhanced the capacity to uptake EXOs and PM-EXOs.

Collectively, PM-EXOs were primarily taken up by endothelial cells and macrophages, and they were able to enhance the cellular uptake and functional miRNA delivery efficiency. As platelets can aggregate with monocytes after vascular injury, we investigated whether PM-EXOs could reach the injured site and traverse the endothelium by hitchhiking on monocytes. As shown in the transwell assay in Figure S4A-C, PM-EXOs alone showed minimal transendothelial migration, whereas the co-incubation with inflammatory monocytes significantly enhanced PM-EXO migration to the lower chamber. Unlike macrophage phagocytosis, PM-EXOs primarily adhered to the monocyte membrane (Figure S5). The adhesion of PM-EXOs did not affect the migratory capacity of inflammatory monocytes (Figure S4D). These results suggested that PM-EXOs can leverage monocyte adhesion to cross the endothelium and enhance tissue permeability. Upon reaching the injured site, monocytes differentiate into macrophages under inflammatory environment and uptake PM-EXOs with their functional miRNAs.

### Enhanced proliferation and migration capacities of endothelial cells by PM-EXOs *in vitro*

**Figure 4:**
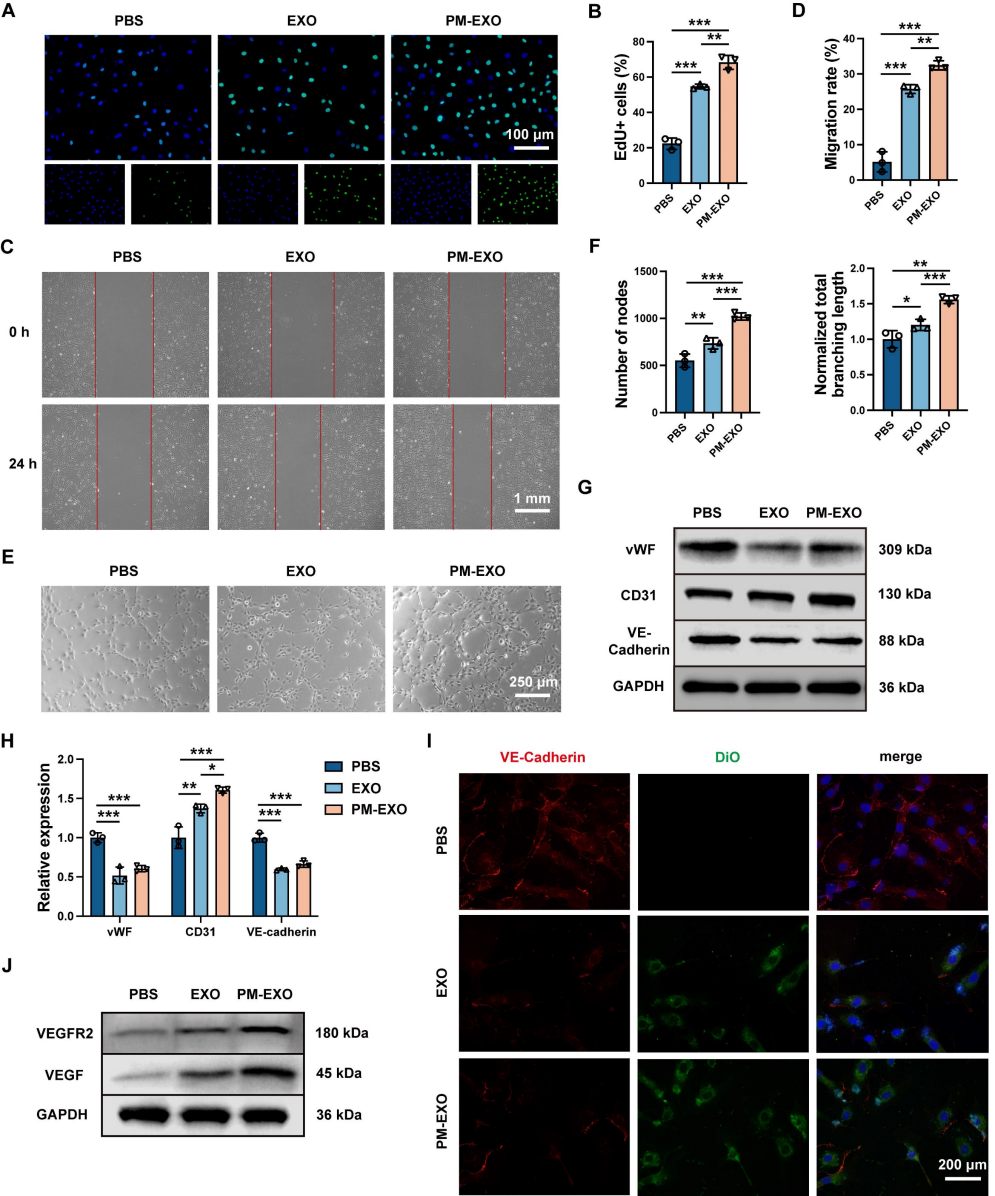
Proangiogenic capability of PM-EXOs toward endothelial cells *in vitro*. (A) Representative fluorescence images of HUVEC proliferation determined by EdU staining. Scale bar = 100 μm. (B) Semi-quantification of the percentage of EdU-positive HUVECs coincubated with PBS, EXOs, or PM-EXOs. (C) Images of the scratch assay and (D) the quantification of the migration rate of HUVECs from 0 h to 24 h. Scale bar = 1 mm. (E) Images of the tube formation assay after 6-h incubation and (F) the quantification of the number of nodes and normalized total branching length of HUVECs. Scale bar = 250 μm. (G) Western blotting analysis of the expression of vWF, CD31, and VE-cadherin in HUVECs after 24-h coincubation with PBS, EXOs, or PM-EXOs and (H) the semi-quantification of their relative expression. (I) Representative fluorescence images of VE-cadherin staining in HUVECs coincubated with DiO-labeled EXOs or PM-EXOs for 24 h. Scale bar = 200 μm. (J) Western blotting of the expression of VEGFR2 and VEGF in HUVECs treated with PBS, EXOs, or PM-EXOs. Data are expressed as the mean ± SD (n = 3, *P < 0.05, **P < 0.01, ***P < 0.001 by one-way ANOVA with a Tukey post hoc test (B, D, F, H)).

The proliferation and migration of endothelial cells are crucial for arterial re-endothelialization. EdU proliferation assays, scratch assays, and tube formation assays were respectively performed to examine the effects of PM-EXOs on the proliferation, migration, and vasculogenic capacities of HUVECs. Both EXOs and PM-EXOs significantly enhanced the proportion of EdU^+^ HUVECs and narrowed the wound in the scratch assays, with this effect being most pronounced in the PM-EXO group (**Figure 4**A-D). In tube formation assays, PM-EXOs induced a 1.4-fold increase in the number of nodes and a 1.3-fold increase in the branching length of HUVECs compared to EXOs (Figure 4E-F). Previous studies have reported that highly proliferative and migratory endothelial cells are characterized by the decreased expression of vWF and VE-cadherin, along with increased expression of CD31 33, 34. The results of western blotting showed that PM-EXOs significantly downregulated the expression of vWF and VE-cadherin while upregulating the expression of CD31 in HUVECs (Figure 4G-H). Immunofluorescence staining further confirmed the reduced VE-cadherin expression in the PM-EXO group (Figure 4I).

Vascular endothelial growth factor (VEGF) is a well-established regulator of physiological angiogenesis, modulating vWF and VE-cadherin expression through the VEGF-VEGFR2 signaling pathway in endothelial cells 33, 34. Proangiogenic miRNAs delivered by PM-EXOs to HUVECs, such as miR-21-5p, miR-126-3p, and miR-486, have all been reported to be involved in the VEGF-VEGFR2 pathway and to promote endothelial cells angiogenesis 35-37. As shown in Figure 4J and Figure S6A, western blotting showed that PM-EXOs increased VEGF and VEGFR2 expression by 1.4-fold compared EXOs, indicating a proangiogenic effect via the VEGF-VEGFR2 pathway. Taken together, these findings demonstrated that PM-EXOs enhanced endothelial cell proliferation and migration, potentially accelerating re-endothelialization at the site of arterial injury *in vivo*.

### Repolarization of activated macrophages and amelioration of inflammatory milieu by PM-EXOs *in vitro*

**Figure 5:**
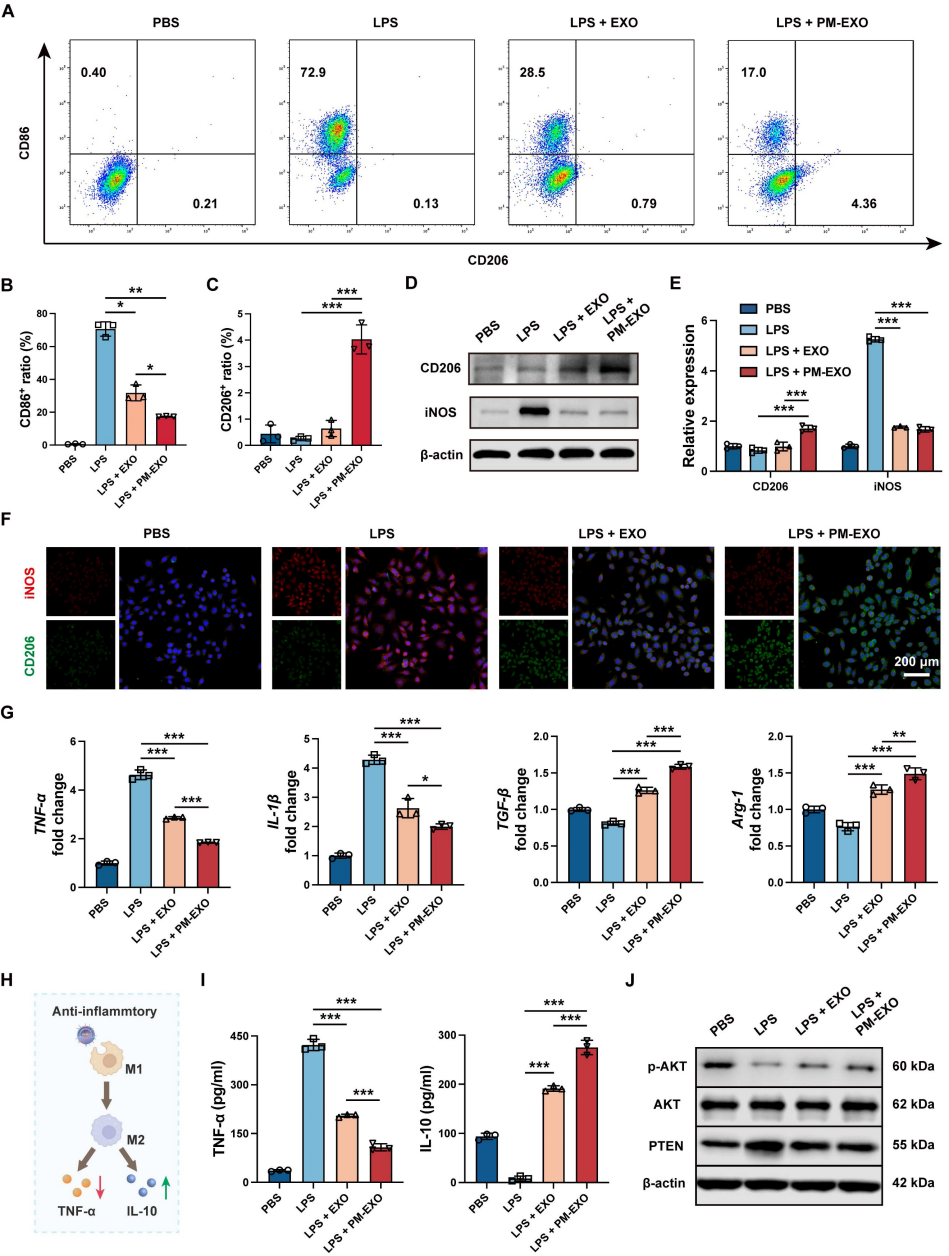
Immunomodulatory capacity of PM-EXOs in macrophages *in vitro*. (A) Representative flow cytometry plots of the proinflammatory M1 phenotype (CD86^+^) and the anti-inflammatory M2 phenotype (CD206^+^) after incubation with PBS, EXOs, or PM-EXOs with or without LPS pretreatment. (B-C) Quantification of the percentage of M1 and M2 macrophages. (D) Western blotting of the expression of M1 marker iNOS and M2 marker CD206 and (E) the semi-quantification of their relative expression. (F) Representative confocal images of iNOS (red) and CD206 (green) double staining in RAW 264.7 cells in the five different groups. Scale bar = 200 μm. (G) Quantification of the expression of M1-related mRNAs (*TNF-α* and *IL-1β*) and M2-related mRNAs (*TGF-β* and *Arg-1*) in RAW 264.7 cells by qRT-PCR. (H) A schematic diagram illustrating how PM-EXOs improve inflammatory melieu by modulating macrophage repolarization. (I) Concentration of proinflammatory cytokine TNF-α and anti-inflammatory cytokine IL-10 in supernatants. (J) Western blotting of the expression of PTEN, p-AKT, and AKT in RAW 264.7 cells treated with PBS, EXOs, or PM-EXOs following LPS pretreatment. Data are expressed as the mean ± SD (n = 3; *P < 0.05, **P < 0.01, ***P < 0.001 by one-way ANOVA with a Tukey post hoc test (B, C, E, G, I)).

As inflammatory response and macrophage infiltration can exaggerate the severity of arterial restenosis, we examined the immunomodulatory capability of PM-EXOs on activated macrophages *in vitro.* As shown in **Figure 5**A, LPS treatment largely increased the proportion of M1 macrophages (CD86^+^), while EXOs and PM-EXOs reversed the M1-phenotypic switch and increased the proportion of M2 macrophages (CD206^+^). Compared to the LPS + EXO group, the LPS + PM-EXO group exhibited a markedly lower proportion of M1 macrophages (31.8% vs. 17.4%; *P* < 0.05) and a higher proportion of M2 macrophages (0.6% vs. 4.0%; *P* < 0.001) (Figure 5B-C). Consistent with the results of flow cytometry, western blotting and immunofluorescence staining revealed that PM-EXO treatment significantly reduced the expression of M1 marker iNOS and raised the expression of M2 marker CD206 (Figure 5D-F). Furthermore, qRT-PCR showed that PM-EXO treatment downregulated the mRNA expression of M1 marker genes (*TNF-α, IL-1β*) and upregulated M2 marker genes (*TGF-β, Arg-1*) (Figure 5G). We also investigated the impact of macrophages on the inflammatory environment in different treatment groups (Figure 5H). After LPS stimulation, RAW 264.7 cells were activated and released typical inflammatory cytokine TNF-α into supernatants and reduced the production of anti-inflammatory cytokine IL-10 (Figure 5I). Conversely, coincubation with PM-EXOs significantly decreased TNF-α concentration and increased IL-10 concentration in supernatants.

Previous studies have reported that the PTEN-AKT signaling pathway is involved in macrophage polarization and that PTEN can block M2 polarization by suppressing AKT activation 38, 39. Accordingly, we investigated the expression of PTEN and p-AKT in RAW 264.7 cells via western blotting. As shown in Figure 5J and Figure S6B, LPS treatment induced PTEN overexpression and suppressed AKT activation, indicated by decreased phosphorylation. Compared to the LPS group, PM-EXO treatment markedly downregulated PTEN expression by 40% and upregulated the p-AKT/AKT ratio by 1.3-fold. Overall, PM-EXO treatment effectively repolarized activated macrophages from the M1 to the M2 phenotype and improved the inflammatory environment.

### Inhibition of migration and phenotypic switch of VSMCs via modulating inflammation by PM-EXOs* in vitro*

**Figure 6:**
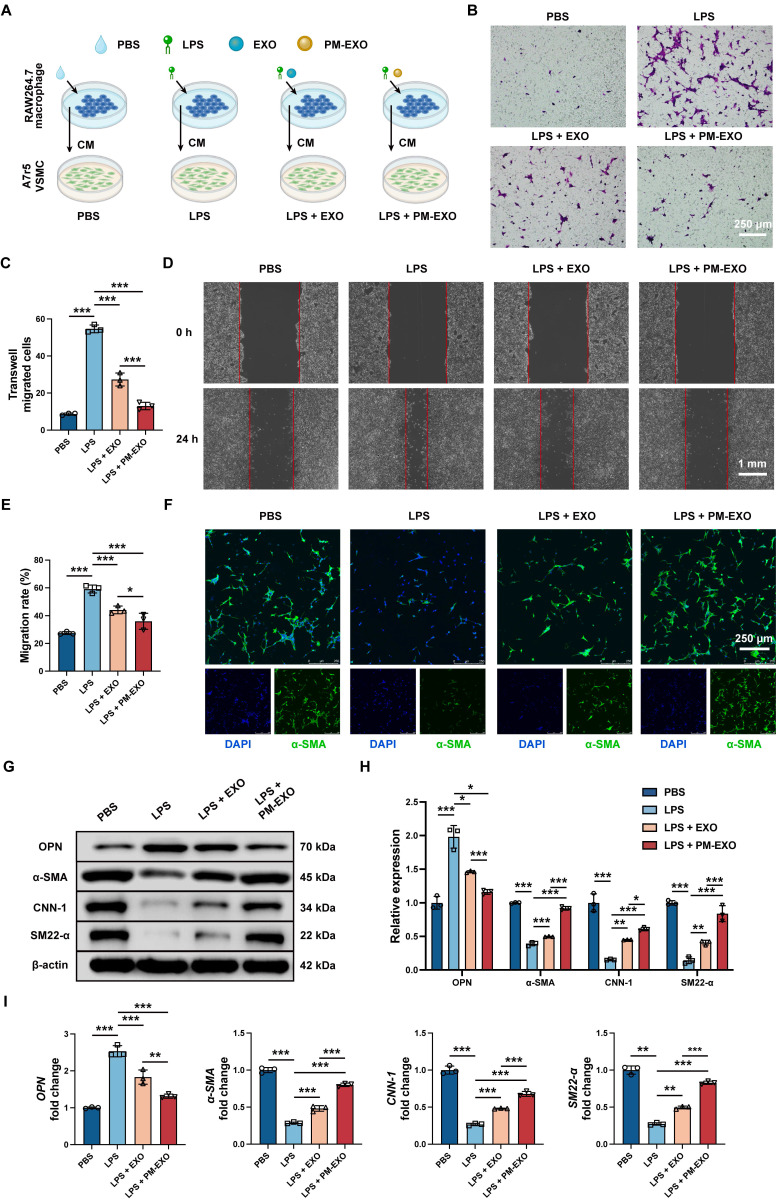
Inhibition of VSMC migration and phenotypic switch by PM-EXOs through immunomodulation *in vitro*. (A) Schematic illustration of experiments demonstrating the indirect effects of PM-EXOs on VSMCs via macrophages modulation and inflammatory environment alteration. RAW 264.7 cells were incubated with PBS, EXOs, or PM-EXOs after LPS pretreatment, and their supernatants were collected for subsequent incubatation with A7r5 cells. (B) Images of the transwell migration assay and (C) the quantification of migrated A7r5 cells after 24-h incubation. Scale bar = 250 μm. (D) Images of the scratch assay and (E) the quantification of the migration rate of A7r5 cells after 24-h incubation. Scale bar = 1 mm. (F) Representative confocal images of α-SMA staining in A7r5 cells in the four different groups. Scale bar = 250 μm. (G) Western blotting results of the expression of contractile VSMC markers (α-SMA, CNN-1 and SM22-α) and synthetic VSMC marker OPN and (H) the semi-quantification of their relative expression. (I) Quantification of *OPN*, *α-SMA*, *CNN-1*, and *SM22-α* mRNA expressions in the four different groups by qRT-PCR. Data are expressed as the mean ± SD (n = 3; *P < 0.05, **P < 0.01, ***P < 0.001 by one-way ANOVA with a Tukey post hoc test (C, E, H, I)).

Neointima hyperplasia is the direct causative factor of arterial restenosis and involves an excessive accumulation of pathological VSMCs. We investigated the potential impact of PM-EXOs on VSMCs by modulating neighboring macrophage polarization and the inflammatory environment. RAW 264.7 cells were treated with PBS, LPS, LPS + EXOs, or LPS + PM-EXOs, and then their supernatants were transferred for coculture with A7r5 cells for 24 h (**Figure 6**A). Transwell and scratch assays were conducted to assess the migration ability of A7r5 cells across different treatments.

As shown in Figure 6B-E, supernatants in the LPS group increased the number of migrated cells and accerlerated wound closure, while PM-EXOs abolished the enhancement of VSMC migration capacity. As for phenotypic switch assessment, immunofluorescence staining for the contractile VSMC marker α-SMA showed prominent green fluorescence of α-SMA in the PM-EXOs group, whereas the LPS groups displayed weak signals (Figure 6F). The results of western blotting and qRT-PCR further demonstrated that the supernatants in the LPS group upregulated the synthetic VSMC marker OPN and downregulated contractile VSMC markers α-SMA, CNN-1, and SM22-α in A7r5 cells (Figure 6G-I). In contrast, the supernatants in the PM-EXOs group evidently recovered the expression of α-SMA, CNN-1, and SM22-α and suppressed OPN expression. Taken together, these results indicated that the inflammatory environment created by activated macrophages stimulated VSMC migration and phenotypic switch, while PM-EXOs inhibited these pathological changes by repolarizing macrophages and ameliorating inflammation.

### Selective targeting of PM-EXOs towards arterial injuries* in vivo*

**Figure 7:**
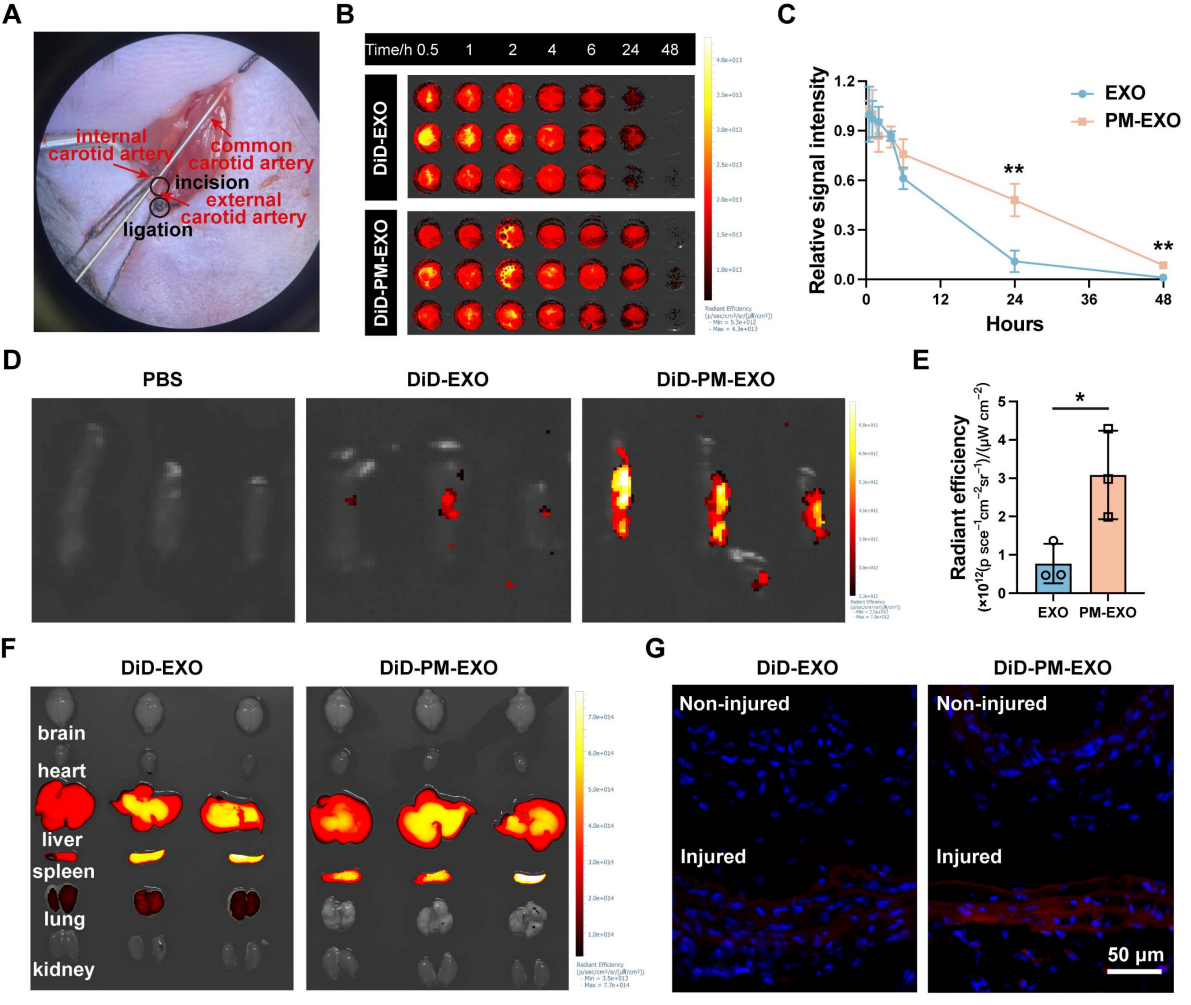
Pharmacokinetics and targeting efficiency of PM-EXOs in a carotid artery wire injury mouse model. (A) Representative image of mouse carotid artery wire injury modeling. (B) *Ex vivo* near-infrared fluorescence (NIRF) imaging of the whole blood collected at various timepoints after intravenous administration of DiD-labeled EXOs or PM-EXOs and (C) the corresponding time-dependent blood circulation curve. (D) *Ex vivo* NIRF imaging of DiD signals accumulating in the injured carotid arteries and (E) semi-quantification of their radiant efficiency. (F) *Ex vivo* NIRF imaging of major organs collected from different groups. (G) Representative images showing traces of EXOs or PM-EXOs (DiD, red) on the endothelium of the non-injured or injured side of carotid arteries. Scale bar = 50 μm. Data are expressed as the mean ± SD (n = 3, *P < 0.05, **P < 0.01, ***P < 0.001 by unpaired two-tailed Student's *t*-test (C, E)).

To examine the *in vivo* targeting ability of PM-EXOs, we established a mouse carotid artery wire injury model as described previously 40 (**Figure 7**A), followed by intravenous injection of PBS, EXOs, or PM-EXOs. Mice were sacrificed according to schedule, and the injured common carotid arteries were isolated for further experimental analysis (Figure S7). For *in vivo* pharmacokinetics, DiD-labeled EXOs or PM-EXOs were intravenously administrated, with blood samples collected at specified time points. As shown in Figure 7B-C, PM-EXOs exhibited a significantly prolonged circulation time compared to EXOs (*P* < 0.01), likely due to the presence of CD47 inherited from the platelet membrane 41. Regarding targeting efficacy, *ex vivo* near-infrared fluorescence (NIRF) imaging on day 1 post-administration showed that the average fluorescence intensity of injured carotid arteries treated with DiD-labeled PM-EXOs was about 4-fold higher than that treated with DiD-labeled EXOs, indicating superior accumulation of PM-EXOs at injury sites (Figure 7D-E). Fluorescence images of the vessel sections corroborated these findings. There was negligible DiD-labeled (red) EXO or PM-EXO deposition on the endothelium of non-injured side of carotid arteries. In contrast, injured arteries in both the EXO and PM-EXO groups exhibited red signal accumulation on the arterial endothelium, with significantly higher fluorescence intensity in the PM-EXO group (Figure 7G). As for the biodistribution in other organs, EXOs and PM-EXOs mainly accumulated in the liver and spleen due to the action of the mononuclear phagocyte system (Figure 7F).

In short, PM-EXOs demonstrated an enhanced ability to actively target injured arteries with prolonged circulation time after systemic administration. Studies have indicated that in addition to direct aggregation at vascular injury sites, platelets in patients with artery disease can also enhance their binding to circulating monocytes and follow them to the injured area 26, 42. To ascertain whether PM-EXOs could target arterial injuries by adhering to monocytes, DiD-labeled EXOs or PM-EXOs were intravenously injected, and the proportion of DiD^+^Ly6C^+^ monocytes in mice blood circulation was measured by flow cytometry. As shown in Figure S8, more PM-EXOs adhered to the Ly6C^+^ monocytes than did EXOs (*P* < 0.01), indicating that PM-EXOs not only directly aggregated at the site of arterial injury but also hitchhiked on inflammatory monocytes to reach the injured area.

### Expedited endothelial repair of injured arteries by PM-EXOs in mouse carotid artery wire injury model

**Figure 8:**
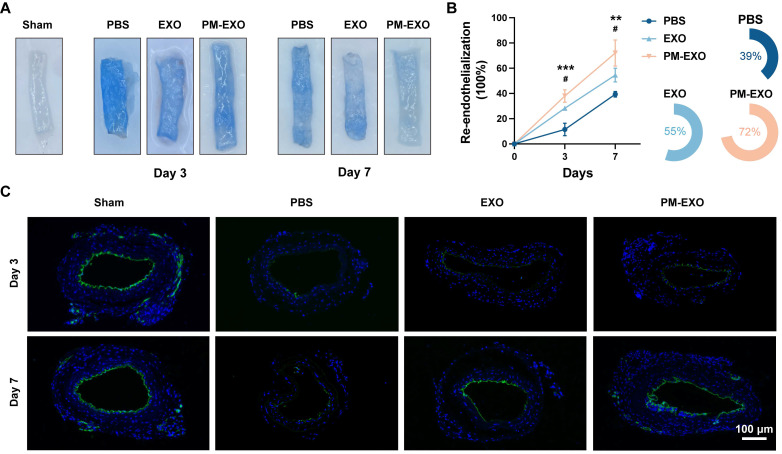
Promoted endothelial repair of the injured arteries by PM-EXOs. (A) Representative images of Evans blue-stained carotid arteries on day 3 and 7 post-injury and (B) semi-quantification of the re-endothelialization rates in different groups (n = 4). (C) Representative fluorescence images of CD31-stained mouse carotid arteries on day 3 and 7 post-injury that reflect the re-endothelialization extent. Scale bar = 100 μm. Data are expressed as the mean ± SD (*/#*P* < 0.05, **/##*P* < 0.01, ***/###*P* < 0.001 by one-way ANOVA with a Tukey post hoc test (B); * compared with the PBS group, # compared with the EXO group).

To investigate the therapeutic advantages of PM-EXOs in re-endothelialization, wire injured mouse carotid arteries were harvested on day 3 and 7 post-administration. As shown in **Figure 8**A, Evans blue staining indicated sever endothelial denudation in the PBS group, which was significantly alleviated in the EXO group and PM-EXO group. Re-endothelialization rates on day 3 showed 11%, 28%, and 40% recovery in the PBS group, EXO group, and PM-EXO group, respectively; by day 7, recovery rates increased to 39%, 55%, and 72%, respectively (Figure 8B). PM-EXOs demonstrated superior efficacy in promoting endothelial repair compared to EXOs (*P* < 0.05), accelerating re-endothelialization by 1.4 times on day 3 and 1.3 times on day 7. Furthermore, immunofluorescence staining of the endothelial cell marker CD31 was performed on artery sections. As shown in Figure 8C, the discontinuous expression of CD31 revealed a severe denudation caused by wire injury in the PBS group, whereas the endothelial integrity was substantially restored in the PM-EXO group, affirming the potential of PM-EXOs to promote re-endothelialization after arterial injury *in vivo*.

### Mitigated inflammation and inhibition of neointima hyperplasia by PM-EXOs *in vivo*

**Figure 9:**
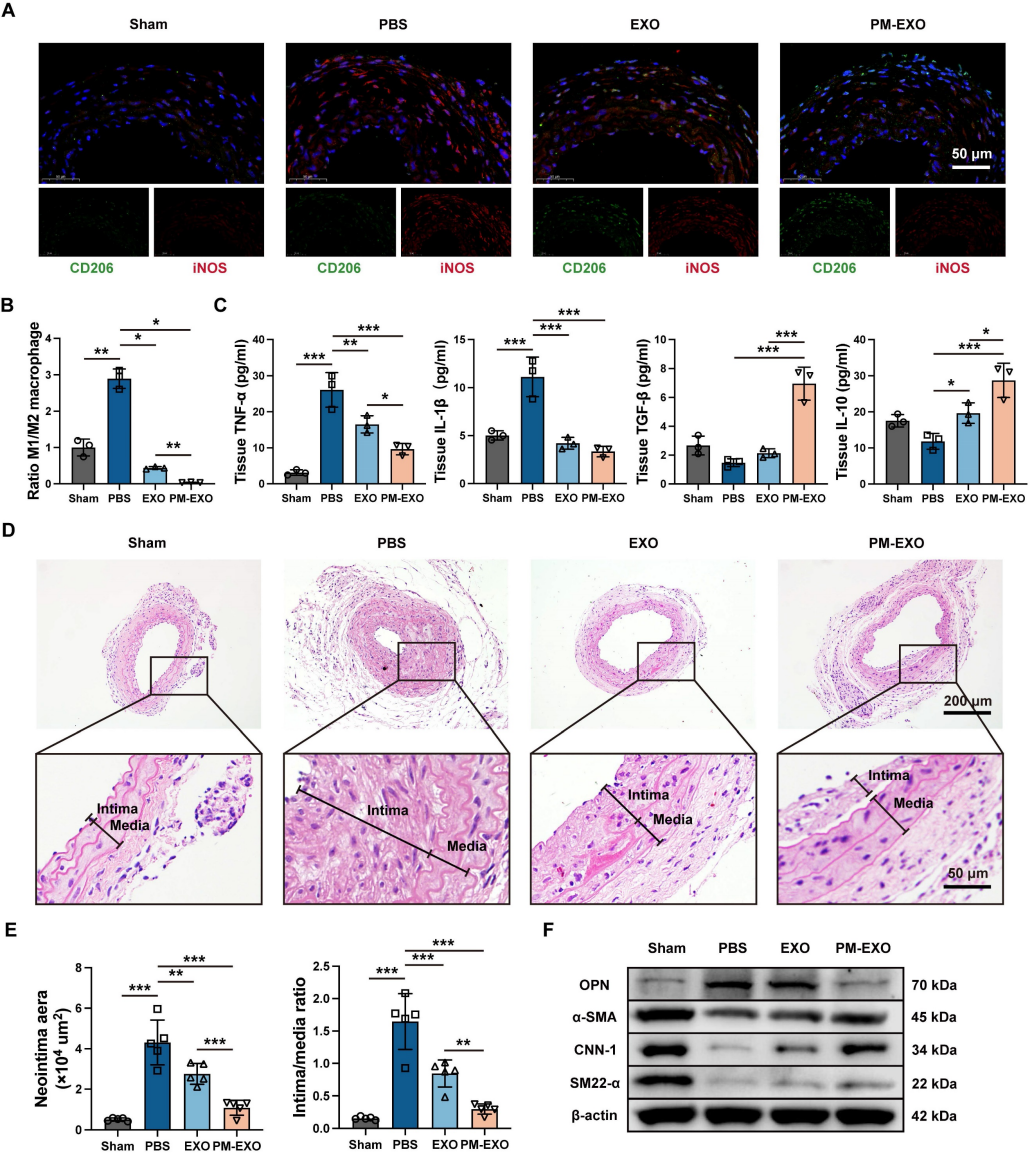
Modulation of inflammatory response and inhibition of neointima hyperplasia in injured arteries by PM-EXOs. (A) Representative fluorescence images of the CD206 and iNOS double-stained carotid arteries harvested on day 3 post-injury and (B) semi-quantification of the ratio of M1 to M2 macrophages in the four groups. Scale bar = 50 μm. (C) ELISA results for the concentrations of proinflammatory cytokines (TNF-α and IL-1β) and anti-inflammatory cytokines (TGF-β and IL-10) in carotid artery homogenate on day 3 post-treatment (n = 3). (D) Representative images of H&E immunostaining of mouse carotid arteries harvested on day 28 post-injury in different groups. Scale bar = 200 μm and scale bar = 50 μm (enlarged). The black line segments indicate the intima and media. (E) Quantification of the neointima area and intima:media ratio in the four different groups (n = 5). (F) Western blotting results for OPN, α-SMA, CNN-1, and SM22-α expression in carotid artery homogenate on day 7 post-injury. Data are expressed as the mean ± SD (*P < 0.05, **P < 0.01, ***P < 0.001 by one-way ANOVA with a Tukey post hoc test (B, C, E)).

To determine the immunomodulatory ability of PM-EXOs *in vivo*, we assessed local inflammation at the injured arteries and systemic inflammatory responses. On day 7 and 28 post-administration, PM-EXOs consistently attenuated the systemic inflammatory response induced by wire injury, evidenced by reduced serum levels of TNF-α and IL-10 (Figure S9A). For local inflammation at the injured site, wire injured mouse carotid arteries were harvested on day 3 for immunofluorescence staining (**Figure 9**A). Wire injury notably increased iNOS expression within the arterial wall in the PBS group compared to the sham group, indicating substantial M1 macrophage infiltration. Meanwhile, the treatment of EXOs or PM-EXOs significantly decreased iNOS expression and increased CD206 expression, indicating a phenotypic switch of infiltrating macrophages from M1 to M2. PM-EXOs demonstrated supierior immunomodulatory capacity compared to EXOs, resulting in a lower M1:M2 ratio (*P* < 0.01) (Figure 9B). Moreover, the production of proinflammatory cytokines (TNF-α and IL-1β) and anti-inflammatory cytokines (TGF-β and IL-10) in arterial tissues on day 3 and 7 was assessed. PM-EXOs significantly downregulated TNF-β and IL-10 concentrations but upregulated TGF-β and IL-10 levels (Figure 9C and Figure S9B).

Next, we investigated the capacity of PM-EXOs to inhibit neointima hyperplasia. Wire injured mouse carotid arteries were harvested on day 28 for H&E immunostaining. As shown in Figure 9D, severe neointima hyperplasia and luminal stenosis were observed in the PBS group. The EXO treatment attenuated the extent of neointima hyperplasia, while the PM-EXO treatment exerted a further inhibitory effect. Quantification of the neointima area and intima:media ratio corroborated theses findings. The PM-EXO group had a 75% reduction in neointima area compared to the PBS group and an intima:media ratio nearly one-fifth that of the PBS group (Figure 9E). To elucidate the phenotypic switch of VSMCs contributing to neointima hyperplasia, the expression of contractile VSMC markers (α-SMA, CNN-1, and SM22-α) and synthetic VSMC marker (OPN) were assessed in wire injured arteries harvested on day 7 and 28 via western blotting (Figure 9F and Figure S10). The artery injury in the PBS group upregulated OPN and downregulated α-SMA, CNN-1, and SM22-α compared to the sham group. In contrast, PM-EXO treatment markedly reversed these changes, indicating that PM-EXOs effectively prevented the phenotypic switch of VSMCs from contractile to synthetic.

Collectively, our data demonstratde that PM-EXOs effectively ameliorated the inflammatory milieu of the arterial wall and inhibited neointima hyperplasia *in vivo*, as it significantly repolarized infiltrated macrophages from M1 to M2 and suppressed the VSMC pathological phenotypic switch in the injured artery*.*

### Verification of PM-EXO targeting and therapeutic effects in rat carotid artery balloon injury model

To validate the targeting ability and therapeutic effects of PM-EXOs, we also established a rat carotid artery balloon injury model as described previously^40^ and intravenously administered PBS, EXOs, or PM-EXOs. The injured carotid artery and vital organs were collected 1 day after the administration of DiD-labeled PM-EXOs. As shown in Figure S11, the fluorescence was primarily concentrated in the injured carotid artery, and there was no significant fluorescence observed in the other organs, indicating the excellent ability of PM-EXOs to target injured artery. On day 14 after administration, the carotid arteries were harvested for further immunostaining analysis. Consistent with the results of the mouse experiments, PM-EXO treatment significantly enhanced the reendothelialization rates of injured arteries (Figure S12). In addition, PM-EXOs reduced the neointima area by 83% and lowered the intima:media ratio to less than one-fifth of that in the PBS group (Figure S13).

### Biosafety of PM-EXOs *in vivo*

Given concerns about the thrombotic risk in the application of nanoparticles, the coagulation function in mice after PM-EXO administration was investigated. There was no significant difference in prothrombin time (PT), activated partial thromboplastin time (APTT), or fibrinogen (FIB) level between the PBS group and PM-EXO group (Figure S14). With respect to organ toxicity, histological analysis revealed no apparent changes in tissue architecture across the different groups one month after administration (Figure S15). Additionally, ELISA analysis of serum levels of general antibodies, immunoglobulin M (IgM) and IgG revealed no significant differences among the PBS group, EXO group, and PM-EXO group, suggesting the absence of an acute immune response against PM-EXOs (Figure S16).

## Conclusion

In our study, we constructed a platelet-mimetic hybrid system by fusing platelet membranes with MSC-derived EXOs and applied it in the treatment of arterial restenosis for the first time. Our data demonstrated that PM-EXOs maintained excellent physiochemical properties and cargo integrity as natural exosomes after the membrane fusion process. The incorporation of platelet membrane significantly enhanced cellular uptake by endothelial cells and macrophages, facilitating efficient delivery of functional miRNAs. In the mouse carotid artery wire injury model, PM-EXOs selectively targeted the injured arteries and penetrated the arterial wall via direct aggregation and attachment to inflammatory monocytes. PM-EXOs also inherited the proangiogenic and immunomodulatory potential of MSC-derived EXOs. After reaching the sites of artery injury, PM-EXOs accerlerated endothelial repair by enhancing endothelial cell proliferation and migration, and mitigated inflammation by repolarizing infiltrated macrophages. This improved inflammatory environment inhibited VSMC migration and phenotypic switch, thereby reducing neointima hyperplasia and arterial restenosis. The targeting ability and therapeutic effects of PM-EXOs were also validated in a rat carotid artery balloon injury model.

In summary, PM-EXOs successfully achieved a synergistic effect of promoting endothelial repair and inhibiting neointima hyperplasia in the targeted treatment of arterial restenosis. This exosome engineering strategy of platelet membrane modification holds significant potential for clinical translation and advancing precision treatment of cardiovascular diseases.

## Supplementary Material

Supplementary figures.

## Abbreviations

APTT: activated partial thromboplastin time
DiD: 1,1-dioctadecyl-3,3,3^,^,3^,^-tetramethylindodicarbocyanine, 4-chlorobenzenesulfonate salt
DiI: 1,1^'^-dioctadcyl-3,3,3^,^,3^,^-tetramethylindocarbocyanine perchlorate
DiO: 3,3-dioctadecyloxacarbocyanine perchlorate
ELISA: enzyme-linked immunosorbent assay
EXO: exosome
FIB: fibrinogen
FRET: Förster resonance energy transfer
H&E: hematoxylin and eosin
IgM: immunoglobulin M
IgG: immunoglobulin G
LPS: lipopolysaccharides
miRNA: microRNA
MSC: mesenchymal stem cell
NIRF: near-infrared fluorescence
NTA: nanoparticle tracking analysis
PMV: platelet membrane vesicle
PM-EXO: platelet-mimetic exosome
PT: prothrombin time
qRT-PCR: quantitative real-time polymerase chain reaction
TEM: transmission electron microscope
VEGF: vascular endothelial growth factor
VSMC: vascular smooth muscle cell
vWF: von Willebrand factor
