# Supplementary material for: Platelet membrane decorated exosomes enhance targeting efficacy and therapeutic index to alleviate arterial restenosis
Source: Theranostics. 2025 Jan 1;15(2):408–27. doi: 10.7150/thno.103747 (PMC11671393; doi:10.7150/thno.103747)
Supplement: Supplementary file 1 — Supplementary figures. [file thnov15p0408s1.pdf]

Supplementary materials

Platelet membrane decorated exosomes enhance targeting efficacy and therapeutic index to alleviate arterial restenosis

Shan Lu[1,2,3#], Ruihan Wang[1,2,3#], Minghao Cai[1,2,3#], Chen Yuan[4], Bin Gao[1,2,3], Daqiao Guo[1,2,3], Yisheng Xu[4], Weiguo Fu[1,2,3*], Xiaohua Yu[5,6*], Yi Si[1,2,3*]

1. Department of Vascular Surgery, Zhongshan Hospital Fudan University, Shanghai, 200032, PR China.
2. Institute of Vascular Surgery, Fudan University, Shanghai, 200032, PR China.
3. National Clinical Research Center for Interventional Medicine, Shanghai, 200032, PR China.
4. State Key Laboratory of Chemical Engineering, East China University of Science and Technology, Shanghai, 200237, PR China
5. Department of Orthopedics, The second Affliated Hospital of Zhejiang University School of Medicine, Hangzhou, 310009, Zhejiang, PR China
6. Key Laboratory of Motor System Disease Research and Precision Therapy of Zhejiang Province, Hangzhou, 310009, Zhejiang, PR China

#Shan Lu, Ruihan Wang and Minghao Cai contributed equally to this work.

*Corresponding authors: Weiguo Fu (fu.weiguo@zs-hospital.sh.cn), Xiaohua Yu (xiaohua.yu@zju.edu.cn), Yi Si (si.yi@zs-hospital.sh.cn).

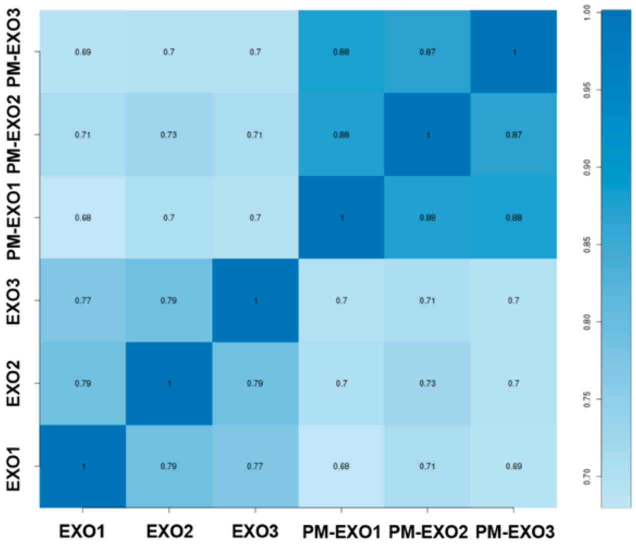

Figure S1. Spearman's correlation analysis of the miRNA profiles of EXOs and PM-EXOs.

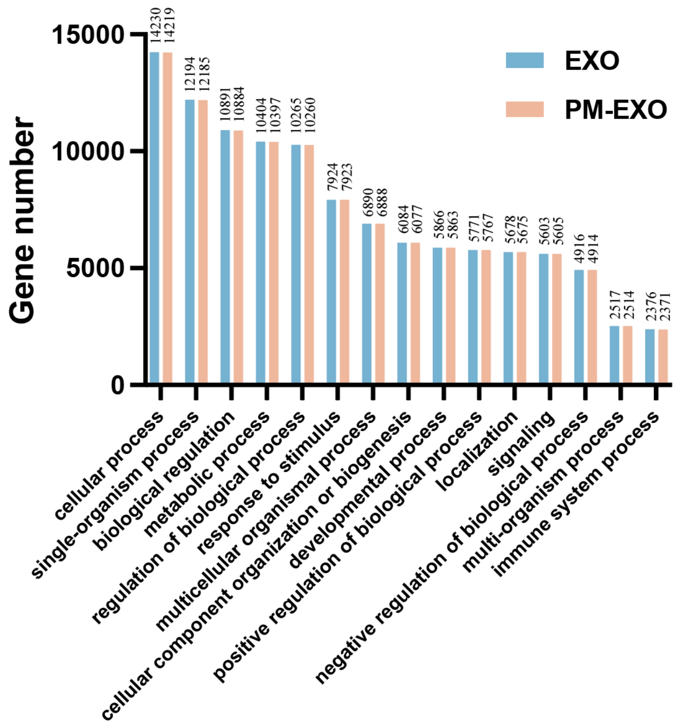

Figure S2. Gene ontology analysis of targeted genes in key biological process in the EXOs and PM-EXOs.

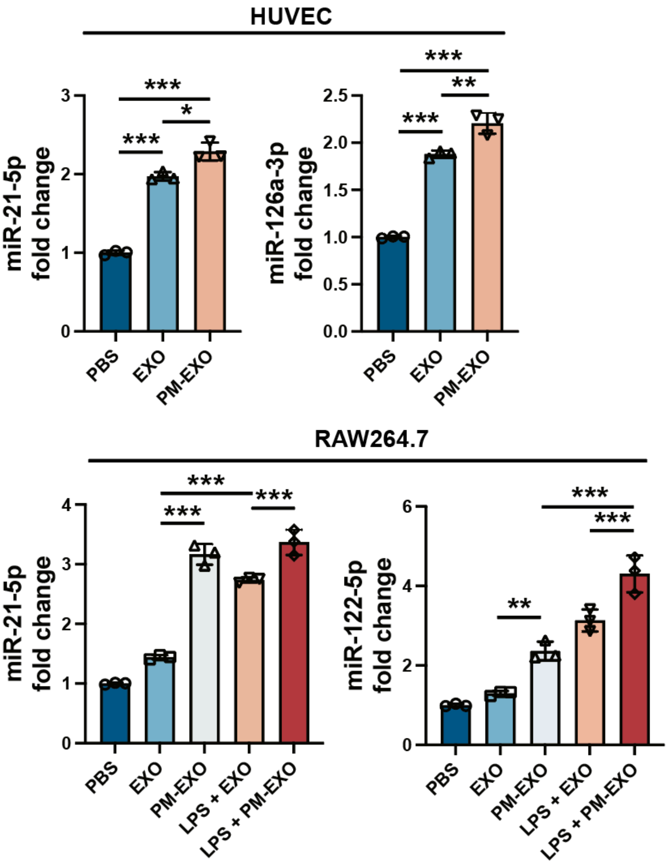

Figure S3. Quantification of major angiogenesis-related miRNAs in HUVECs and immunomodulation-related miRNAs in RAW264.7 cells after coincubation with EXOs or PM-EXOs for 24 h. Data are expressed as the mean ± SD (n = 3, **P* < 0.05, ***P* < 0.01, ****P* < 0.001 by one-way ANOVA with a Tukey post hoc test).

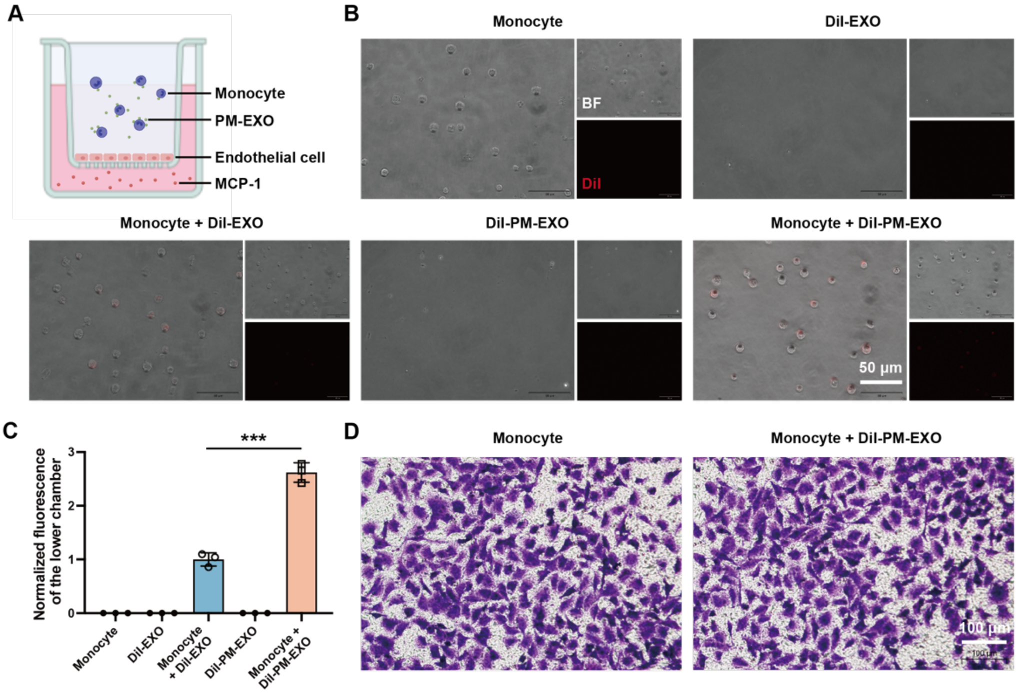

Figure S4. Ability of PM-EXOs to traverse endothelium by monocyte adhesion. (A) Schematic illustration of transwell assay. (B) Representative images and (C) semi-quantification of DiI-labeled EXOs or PM-EXOs (red) on monocytes in the lower chamber after migration through the endothelium. Scale bar = 50 μm. (D) Bright field images of the crystal violet-stained transwell inserts to view the migrated THP-1 cells captured by HUVECs. Scale bar = 100 μm. Data are expressed as the mean ± SD (n = 3, ****P* < 0.001 by unpaired two-tailed Student's *t*-test).

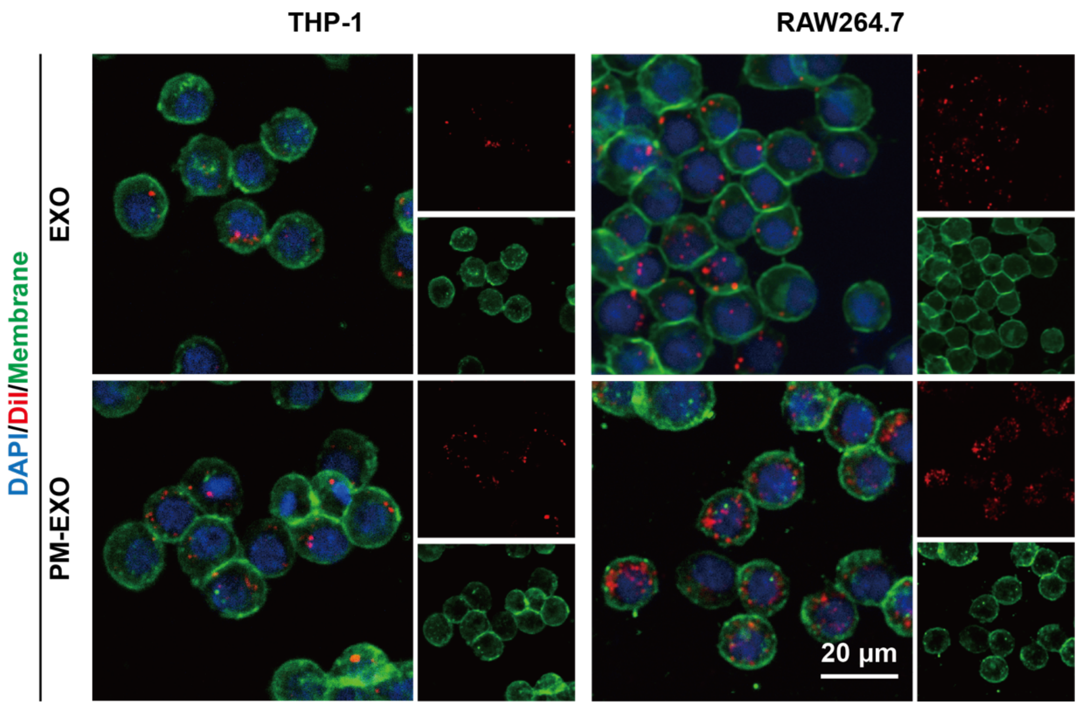

Figure S5. Representative confocal images of THP-1 monocytes and RAW264.7 macrophages co-incubated with DiI-labeled EXOs or PM-EXOs. Scale bar = 20 µm.

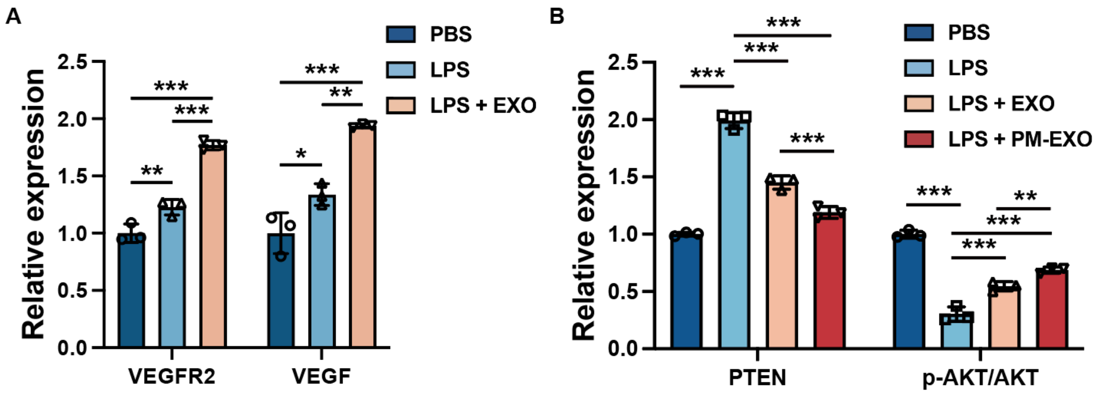

Figure S6. (A) Semi-quantification of VEGFR2 and VEGF expression in HUVECs after 24-h incubation with PBS, EXOs or PM-EXOs (n = 4). (B) Semi-quantification of PTEN expression and activation of AKT in Raw264.7 cells after 24-h incubation with PBS, EXOs or PM-EXOs following LPS pretreatment (n = 3). Data are expressed as the mean ± SD (**P* < 0.05, ***P* < 0.01, ****P* < 0.001 by one-way ANOVA with a Tukey post hoc test (A, B)).

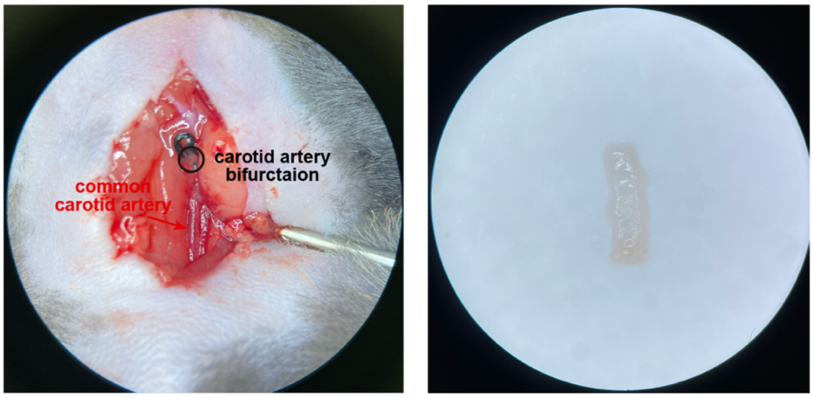

Figure S7. Representative images of the harvested injured common carotid artery.

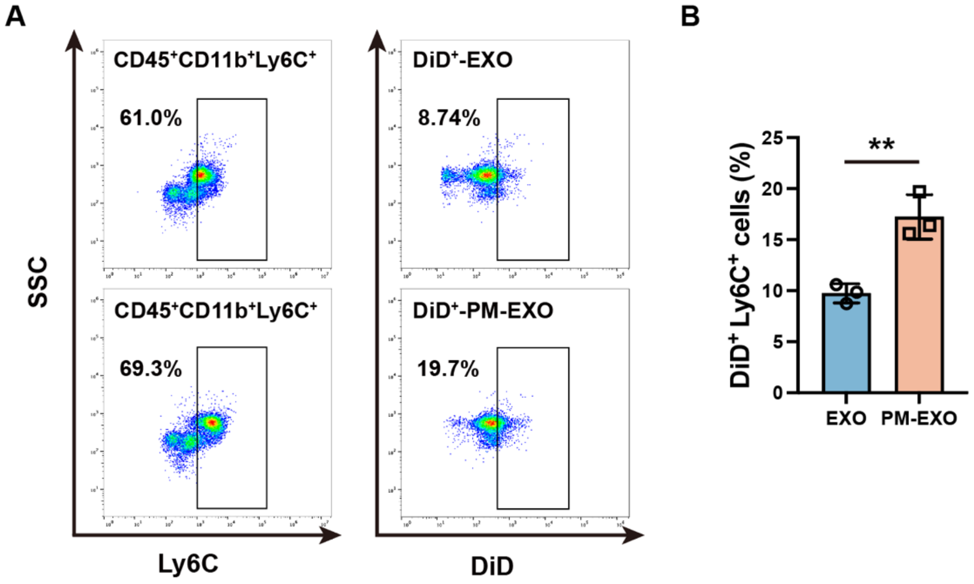

Figure S8. (A) Flow cytometric analysis of the binding ability of PM-EXOs to Ly6C^{+} monocytes in the blood circulation 24 h after arterial injury and (B) quantification of the percentage of DiD^{+}Ly6C^{+} cells (n = 3). Data are expressed as the mean ± SD (*P < 0.05, **P < 0.01, ***P < 0.001 by unpaired two-tailed Student's t-test (B)).

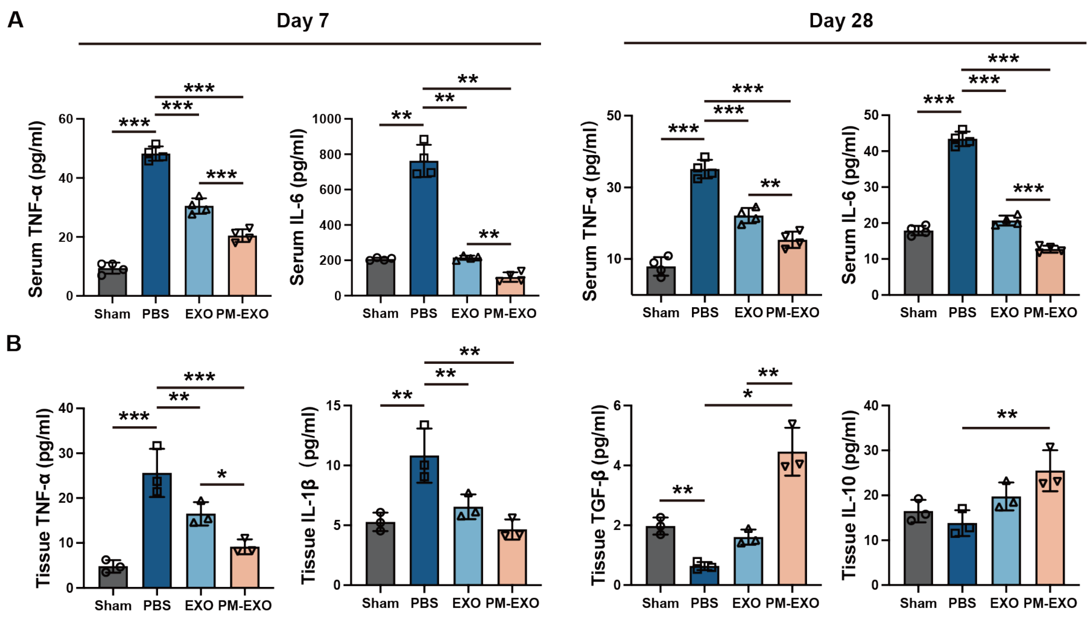

Figure S9. (A) ELISA analysis of TNF-α and IL-6 concentrations in serum on day 7 and day 28 after drug administration that reflects the systemic inflammation level in mice (n = 4). (B) ELISA analysis of the concentrations of pro-inflammatory cytokines (TNF-α and IL-1β) and anti-inflammatory cytokines (TGF-β and IL-10) in carotid artery homogenate on day 7 after drug administration (n = 3). Data are expressed as the mean ± SD (**P* < 0.05, ***P* < 0.01, ****P* < 0.001 by one-way ANOVA with a Tukey post hoc test (A, B)).

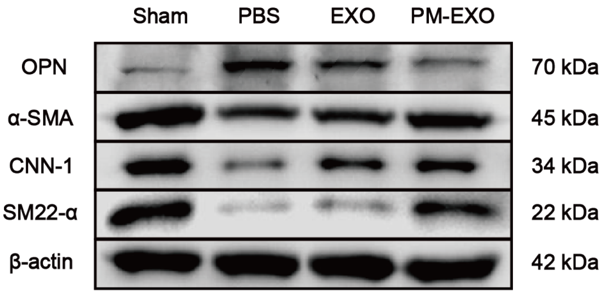

Figure S10. Western blotting analysis of OPN, α-SMA, CNN-1 and SM22-α expression in carotid artery homogenate on day 28 after arterial injury.

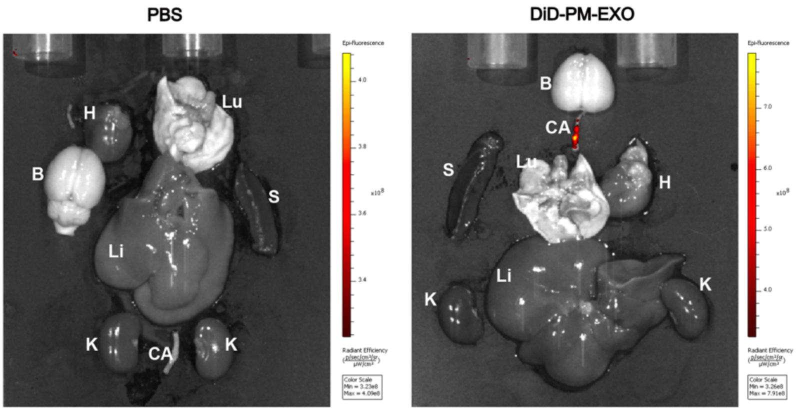

Figure S11. *Ex vivo* near NIRF imaging of the injured carotid artery and other vital organs in the rat carotid artery balloon injury model on day 1 after the intravenous administration of PBS or DiD-labeled PM-EXOs (B: brain, CA: carotid artery, H: heart, K: kidney, Li: liver, Lu: lung, S: spleen).

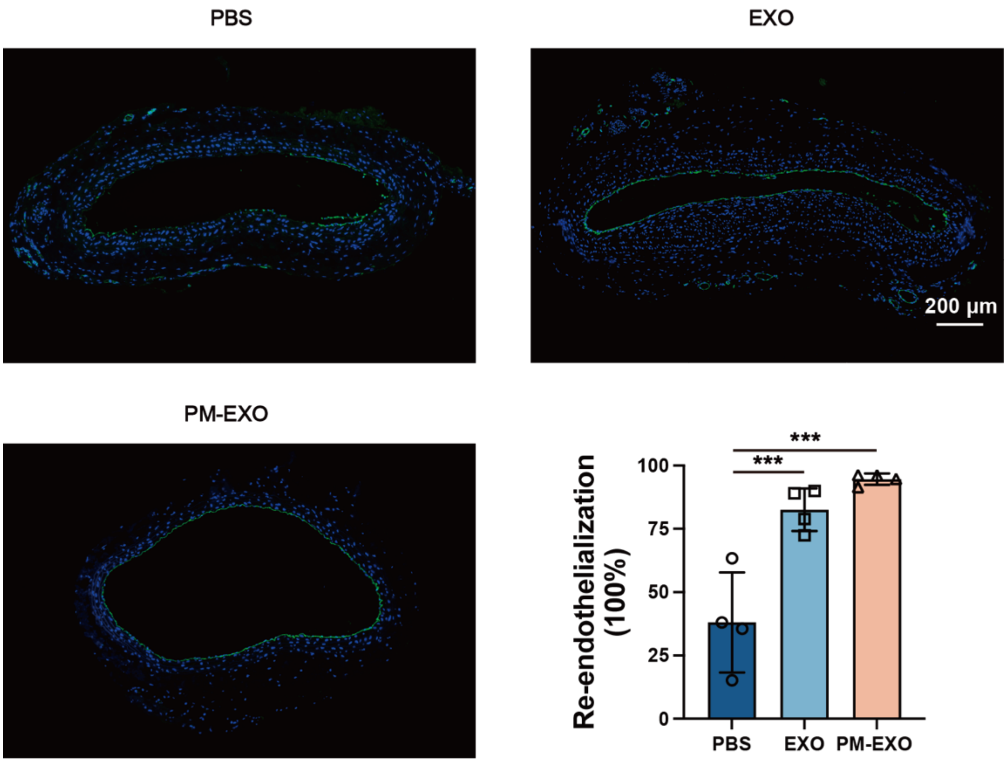

Figure S12. Representative fluorescence images of CD31 stained rat carotid arteries in the different groups harvested on day 14 after injury and semi-quantification of the re-endothelialization rates (n = 4). Scale bar = 200 μm. Data are expressed as the mean ± SD (**P* < 0.05, ***P* < 0.01, ****P* < 0.001 by one-way ANOVA with a Tukey post hoc test).

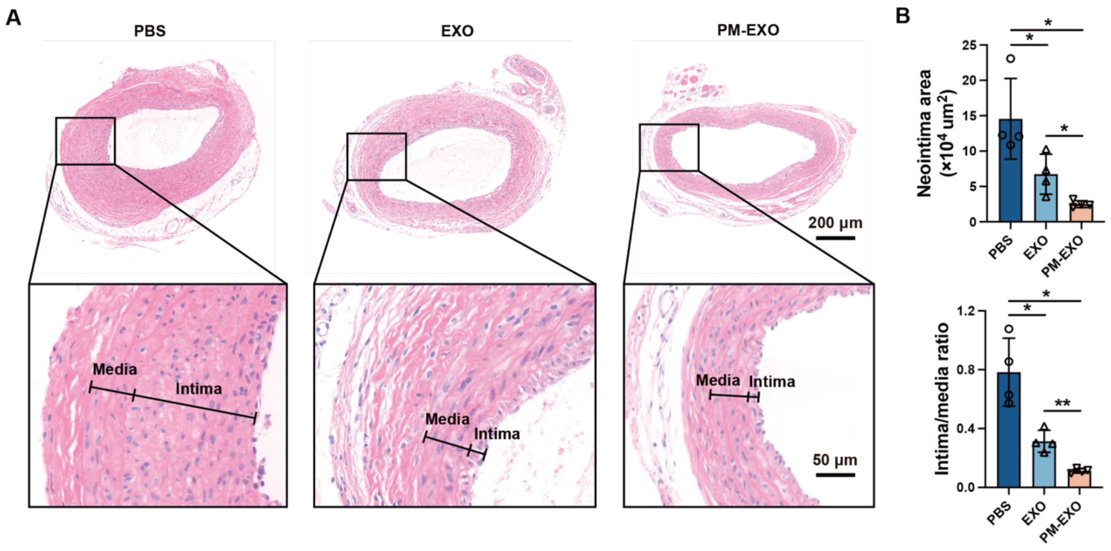

Figure S13. Inhibition of neointima hyperplasia by PM-EXOs in the rat carotid artery balloon injury model. (A) Representative images of H&E immunostaining of rat carotid arteries harvested on day 14 after arterial injury and following treatment with PBS, EXOs or PM-EXOs. Scale bar = 200 µm and scale bar = 50 µm (enlarged). The black line segments indicate the intima and media. (B) Quantification of the neointima area and the intima/media ratio (n = 4). Data are expressed as the mean ± SD (*$P < 0.05$, **$P < 0.01$, ***$P < 0.001$ by one-way ANOVA with a Tukey post hoc test (B)).

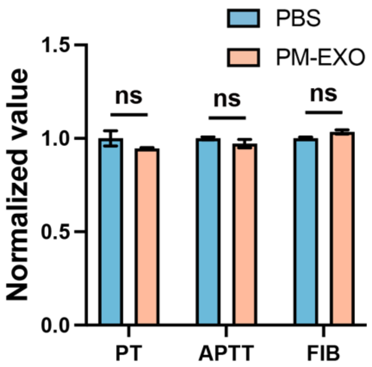

Figure S14. Coagulation function level in mice after drug administration of PBS or PM-EXOs. Data are expressed as the mean ± SD (ns: $P > 0.05$ by one-way ANOVA with a Tukey post hoc test).

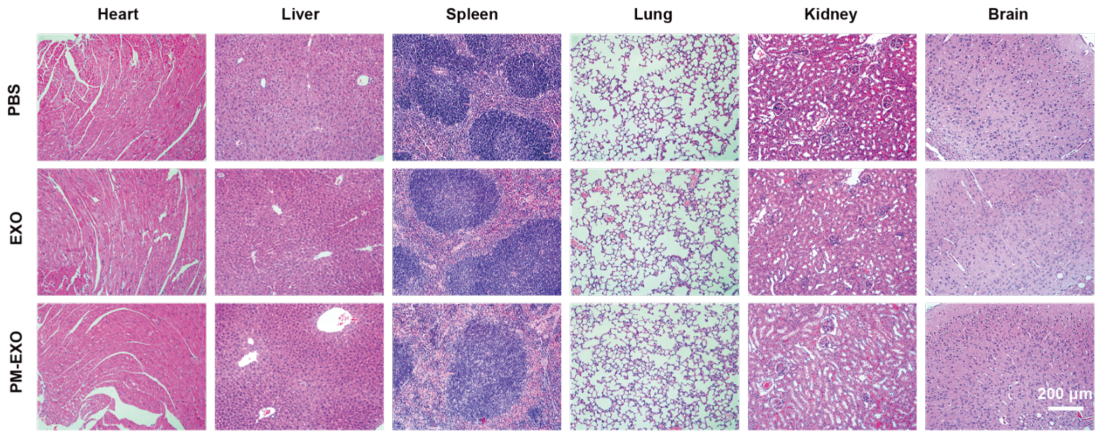

Figure S15. Representative H&E immunostaining images of vital organs in the PBS, EXOs and PM-EXOs group. Scale bar = 200 μm.

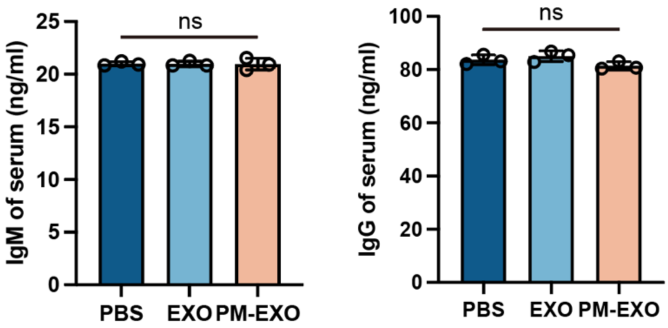

Figure S16. Quantification of the serum levels of general antibodies IgM and IgG by ELISA assay after drug administration of PBS, EXOs or PM-EXOs. Data are expressed as the mean ± SD (ns: $P > 0.05$ by one-way ANOVA with a Tukey post hoc test).
